# *Lactobacillus rhamnosus* and *Bifidobacterium longum* alleviate colitis and cognitive impairment in mice by regulating IFN-γ to IL-10 and TNF-α to IL-10 expression ratios

**DOI:** 10.1038/s41598-021-00096-x

**Published:** 2021-10-19

**Authors:** Xiaoyang Ma, Yoon-Jung Shin, Hyo-Min Jang, Min-Kyung Joo, Jong-Wook Yoo, Dong-Hyun Kim

**Affiliations:** Neurobiota Research Center, College of Pharmacy, Kyung Hee University, 26, Kyungheedae-ro, Dongdaemun-gu, Seoul, 02447 Korea

**Keywords:** Immunology, Microbiology

## Abstract

Gut lactobacilli and bifidobacteria on the immune homeostasis. Therefore, to understand the mechanism in vivo, we selected human fecal *Lactobacillus rhamnosus* NK210 and *Bifidobacterium longum* NK219, which strongly suppressed the IFN-γ to IL-10 expression (IIE) ratio in lipopolysaccharide-stimulated macrophages. Thereafter, we examined their effects on the endotoxin, antibiotics, or antitumor drug-stimulated immune imbalance in mice. Intraperitoneal injection of lipopolysaccharide and oral gavage of ampicillin increased IFN-γ and TNF-α expression in the spleen, colon, and hippocampus, while IL-10 expression decreased. However, intraperitoneal injection of cyclophosphamide suppressed IFN-γ, TNF-α, and IL-10 expression. LPS exposure induced splenic natural killer cell cytotoxicity against YAC-1 cells (sNK-C) and peritoneal macrophage phagocytosis against *Candida albicans* (pMA-P) activities, while cyclophosphamide and ampicillin treatments suppressed sNK-C and pMA-P activities. However, LPS, ampicillin, cyclophosphamide all increased IIE and TNF-α to IL-10 expression (TIE) ratios. Oral administration of NK210 and/or NK219 significantly reduced LPS-induced sNK-C, pMA-P, and IFN-γ expression, while cyclophosphamide- or ampicillin-suppressed sNK-C and pMA-P activities, cyclophosphamide-suppressed IFN-γ, TNF-α, and IL-10 expression, and ampicillin-suppressed IL-10 expression increased. Nevertheless, they suppressed LPS-, ampicillin-, or cyclophosphamide-induced IIE and TIE ratios, cognitive impairment, and gut dysbiosis. In particular, NK219, but not NK210, increased the IIE expression ratio in vitro and in vivo, and enhanced sNK-C and pMA-P activities in normal control mice, while cognitive function and gut microbiota composition were not significantly affected. These findings suggest that NK210, Lactobacillus sp, and NK219, Bifidobacterium additively or synergistically alleviate gut dysbiosis, inflammation, and cognitive impairment with immune imbalance by controlling IIE and TIE ratios.

## Introduction

The immune system consists of innate and adaptive immune cells, of which > 70% are located in the gut^1,2^. Innate immune system is a first-line defense barrier against pathogens, which largely depends on the number of immune cells such as macrophages and natural killer (NK) cells. The activation of innate immune cells by pathogens and their byproducts stimulates the secretion of proinflammatory and anti-inflammatory cytokines such as tumor necrosis factor (TNF)-α, interleukin (IL)-10, and interferon (IFN)-γ, which promote innate and adaptive immune systems^3,4^. In particular, these cytokines stimulate the differentiation of naïve T cells into activated T cells such as helper T (Th) and regulatory T (Treg) cells^5,6^. An imbalance of the immune cytokine expression in innate and adaptive immune cells causes immunosuppressive and hyperresponsive disorders such as autoimmune, chronic inflammatory, infection diseases, and cancers^7,8^. Therefore, regulating the expression of immune cytokines can be useful for the therapy of immune disorders.

Gut microbiota and their byproducts are closely connected with enteric immune and nervous systems, which propagate into other organs in the body by regulating the secretion of cytokines and adrenal hormones^9,10^. Gut dysbiosis is closely associated with gastrointestinal and psychiatric disorders^7,11^. Of gut bacteria, bifidobacteria and lactobacilli have various physiological effects such as immune homeostasis and alleviation against diabetes, colitis, and psychiatric disorder through the direct and/or indirect regulation of gut microbiota and immune and nervous systems^12–14^. For instance, TNF-α expression-suppressing *Lactobacillus mucosae* NK41 alleviates *Escherichia coli*-induced cognitive impairment with gut inflammation in mice^15^. TNF-α expression-inducing *Lactobacillus reuteri* enhances the immune response including IgG expression in mice^16^. IFN-γ expression-inducing *Bifidobacterium bifidum* alleviates influenza infection in mice^17^. IL-10 expression-inducing probiotics alleviates high-fat diet-induced obesity with colitis in mice^18^. IL-10 expression-inducing *Bifidobacterium longum* CECT 7347 alleviates a gliadin-induced enteropathy in rats^19,20^. *Lactobacillus johnsonii* and *Lactobacillus paracasei* PS23 increase hippocampal TNF-α expression in mice with *Escherichia coli*-induced colitis and with age-dependent cognitive decline, respectively^21,22^. *Bifidobacterium longum* NK46 suppresses cognitive decline and hippocampal TNF-α expression in aged and 5XFAD transgenic mice^23^. These results suggest that gut bifidobacteria and lactobacilli can alleviate imbalanced immune responses with psychiatric disorders such as cognitive impairment by the regulation of proinflammatory and/or anti-inflammatory cytokine expression. Nevertheless, how gut lactobacilli and bifidobacteria regulate immune response in vivo under the inflamed or immunosuppressed condition has been not studied sufficiently.

Therefore, to understand how gut bifidobacteria and lactobacilli could singly and mutually regulate the immune response in vivo, we selected *Lactobacillus rhamnosus* NK210 and *Bifidobacterium longum* NK219 from human fecal bacteria collection. Thereafter, we examined the effects of NK210, NK219, and their mixture (Mx) on LPS-generated inflammation, ampicillin-induced gut dysbiosis, and cyclophosphamide-generated immunosuppression in mice.

## Results

### NK210 and NK219 suppressed the IFN-γ to IL-10 expression ratio in LPS-stimulated macrophages

**Figure 1:**
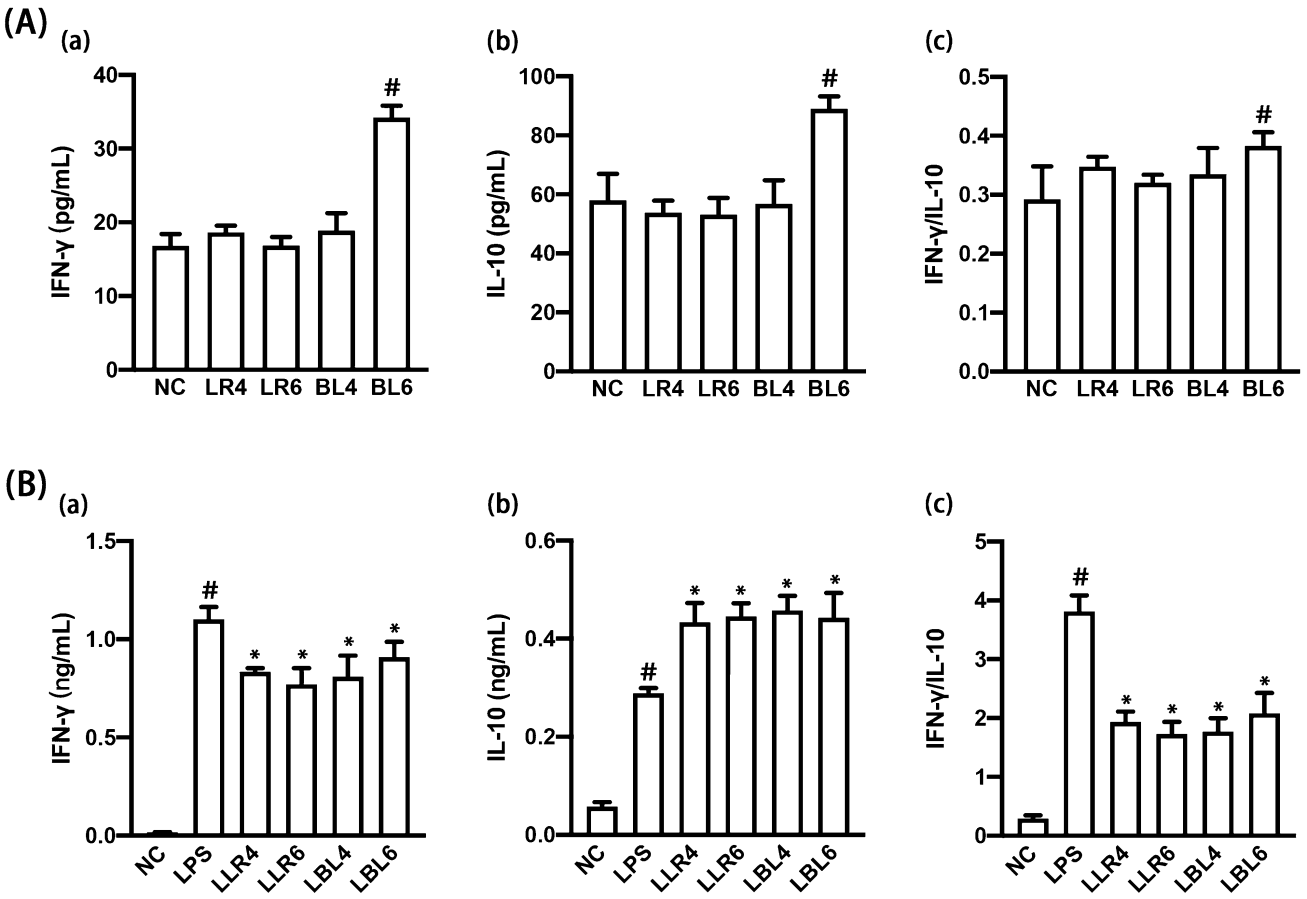
Effect of NK210 and NK219 on the expression of interferon (IFN)-γ and IL-10 in macrophages stimulated with or without LPS. (**A**) Effect on IFN-γ (a) and IL-10 expression (b) and IFN-γ to IL-10 expression ratio (c) in resident macrophage cells. (**B**) Effect on IFN-γ (a) and IL-10 expression (b) and IFN-γ to IL-10 expression ratio (c) in LPS-stimulated macrophage cells. Macrophage cells (1 × 10^6^/mL) isolated from peritoneal cavity were incubated with *Lactobacillus rhamnosus* NK210 (LR4, 1 × 10^4^ CFU/mL NK210; LR6, 1 × 10^5^ CFU/mL) or *Bifidobacterium longum* NK219 (BL4, 1 × 10^4^ CFU/mL NK210; BL6, 1 × 10^5^ CFU/mL) in the absence or presence of LPS. Normal control group (CON) was treated with saline instead of LPS. Data values were described as mean ± SD (n = 4). Data values indicate mean ± SD. ^#^*p* < 0.05 versus NC group. *p < 0.05 versus LP group.

First, we isolated IFN-γ expression-suppressing NK210 and NK219 in LPS-stimulated peritoneal macrophages from human fecal bacteria collection. NK210 and NK219 at doses of 1 × 10^4^ and 1 × 10^6^ CFU/mL strongly suppressed IFN-γ expression in LPS-stimulated peritoneal macrophages (Fig. 1). However, they increased IL-10 expression. They suppressed LPS-induced IFN-γ to IL-10 expression ratio. NK219, not NK210, at a dose of 1 × 10^6^ CFU/mL induced IFN-γ and IL-10 expression in resident macrophages, resulting in the increase in the IFN-γ to IL-10 expression ratio. NK210 and NK219 were identified as *Lactobacillus rhamnosus* and *Bifidobacterium longum* on the basis of the results of Gram staining, API 50 CHL kit, and 16S rRNA gene sequencing (Supplementary Figure S1).

### Effects of NK210 and NK219 on LPS-induced systemic inflammation and cognitive impairment in mice

**Figure 2:**
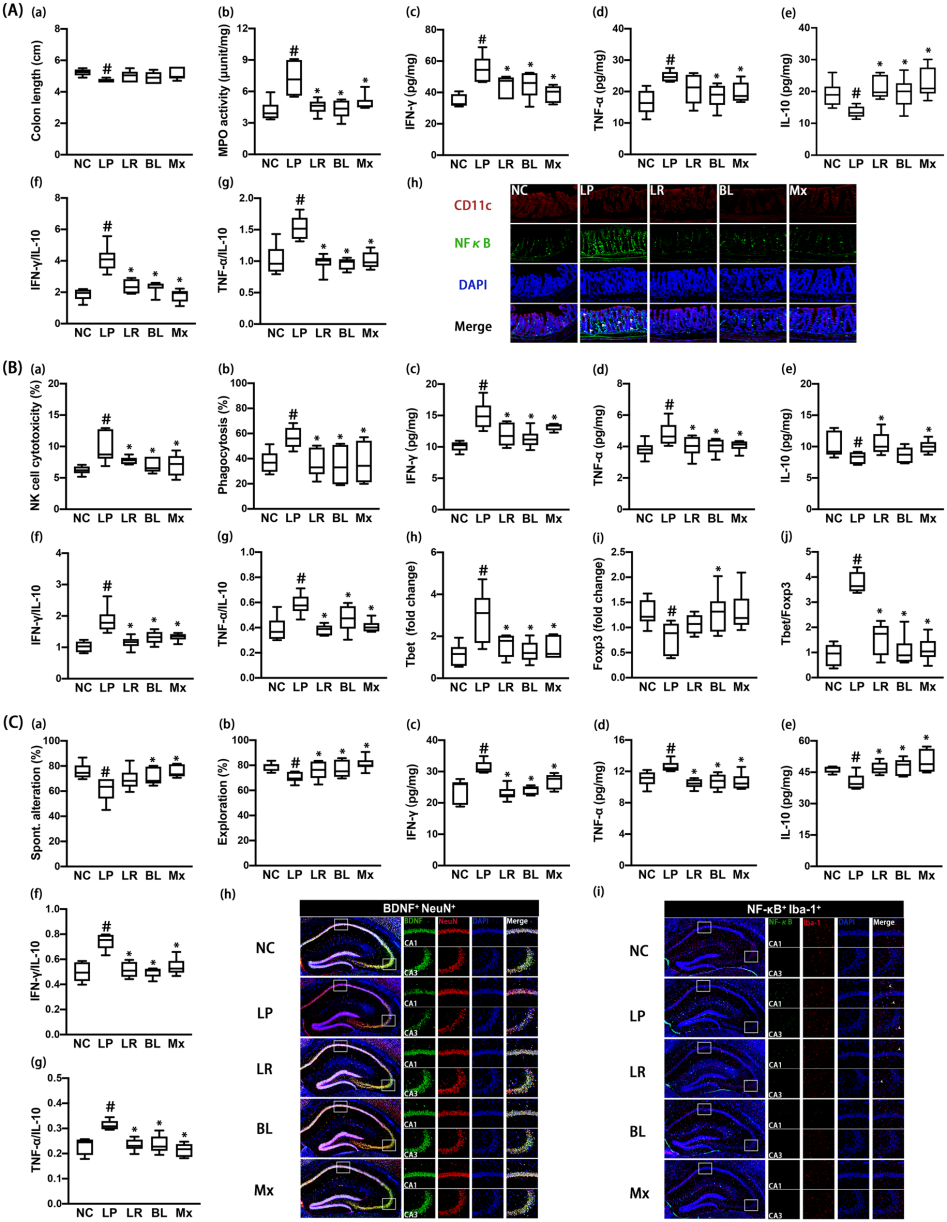
Effects of NK210 and NK219 on LPS-induced immune imbalance and cognitive impairment in mice. (**A**) Effects on colitis: colon length (a), myeloperoxidase (MPO) activity (b), IFN-γ (c), TNF-α (d), IL-10 expression (e), IFN-γ to IL-10 expression ratio (f), TNF-α to IL-10 expression ratio (g), and NF-κB^+^/CD11c^+^ cell population in the colon (h). (**B**) Effects on the immune imbalance: splenic NK cell cytotoxicity activities (a), peritoneal macrophage phagocytosis (b), IFN-γ (c), TNF-α, (d), and IL-10 expression (e), IFN-γ to IL-10 expression ratio (f), TNF-α to IL-10 expression ratio (g), Tbet (h) and Foxp3 expression (i), and Tbet to Foxp3 expression ratio (j) in the spleen. (**C**) Effects on cognitive impairment in the Y-maze (a) and NOR task (b). Effects on neuroinflammation: IFN-γ (c), TNF-α (d), IL-10 expression (e), IFN-γ to IL-10 expression ratio (f), TNF-α to IL-10 expression ratio (g), and BDNF^+^/NeuN^+^ and NF-κB^+^/Iba1^+^ cell populations (h) in the hippocampus. Test agents (LP, LPS alone; LR, 1 × 10^9^ CFU/mouse/day of NK210; BL, 1 × 10^9^ CFU/mouse/day of NK219; Mx, 1 × 10^9^ CFU/mouse/day of LR and BL [4:1] mix) were orally gavaged daily for 5 days after intraperitoneal injection of LPS. Normal control mice (NC) were treated with vehicle (saline) instead of test agents. Data values indicate mean ± SD (n = 8). ^#^*p* < 0.05 versus NC group. *p < 0.05 versus LP group treated with LPS alone.

Intraperitoneal injection of LPS causes cognitive impairment with systemic inflammation in mice^24^. Therefore, we examined effects of NK210 and NK219 on LPS-induced systemic inflammation and cognitive impairment in mice. Intraperitoneal injection of LPS caused colon shortening, increased myeloperoxidase activity, IFN-γ and TNF-α expression, and NF-κB^+^/CD11c^+^ cell population, and suppressed IL-10 expression in the colon (Fig. 2A). In particular, LPS treatment increased IFN-γ to IL-10 and TNF-α to IL-10 expression ratios. However, oral gavage of NK210 or NK219 suppressed LPS-induced colitis: they decreased myeloperoxidase activity, NF-κB^+^/CD11c^+^ cell population, and IFN-γ to IL-10 and TNF-α to IL-10 expression ratios in the colon while colon shortening was not significantly increased. To understand whether NK210 and NK219 antagonistically or synergistically regulate the immune homeostasis, we combined them and examined their effects on the colitis. Of these mixtures, the (4:1) combination synergistically suppressed LPS-induced inflammatory responses in the colon. In particular, it decreased the IFN-γ to IL-10 expression ratio more strongly than NK210 or NK219.

Intraperitoneal injection of LPS also increased the NK cell cytotoxicity against YAC-1 tumor cells (sNK-C) and peritoneal macrophage phagocytic activity against *Candida albicans* (pMA-P) (Fig. 2B). Furthermore, LPS increased IFN-γ, TNF-α, and Tbet expression in the spleen, while the IL-10 and Foxp3 expression decreased. However, oral administration of NK210, NK219, or their (4:1) combination (Mx) suppressed sNK-C and pMA-P activities. They also suppressed IFN-γ and Tbet (a Th1 transcription factor) expression in the spleen. NK210 and Mx, not NK219, increased IL-10 expression. Foxp3 (a Treg cell transcription factor) expression was increased by oral gavage of NK219, not NK210. Nevertheless, they suppressed IFN-γ to IL-10, TNF-α to IL-10, and Tbet to Foxp3 expression ratios in the spleen.

LPS treatment also increased cognitive impairment-like behaviors in the Y-maze and novel object recognition (NOR) tasks (Fig. 2C, Supplementary Figure S2). It increased IFN-γ and TNF-α expression and NF-κB^+^/Iba1^+^ cell population in the hippocampus, while the BDNF^+^/NeuN^+^ cell population and IL-10 expression decreased. However, oral administration of NK210, NK219, or Mx decreased cognitive impairment-like behaviors, NF-κB^+^/Iba1^+^ cell population, and TNF-α and IFN-γ expression in the hippocampus, while BDNF^+^/NeuN^+^ cell population and IL-10 expression increased. There were no significant differences between the cognitive impairment-ameliorating effects of Mx and NK210 or NK219. They also suppressed IFN-γ to IL10 and TNF-α to IL-10 expression ratios in the brain.

### Effects of NK210 and NK219 on cognitive impairment and gut inflammation in mice with ampicillin-induced gut dysbiosis

**Figure 3:**
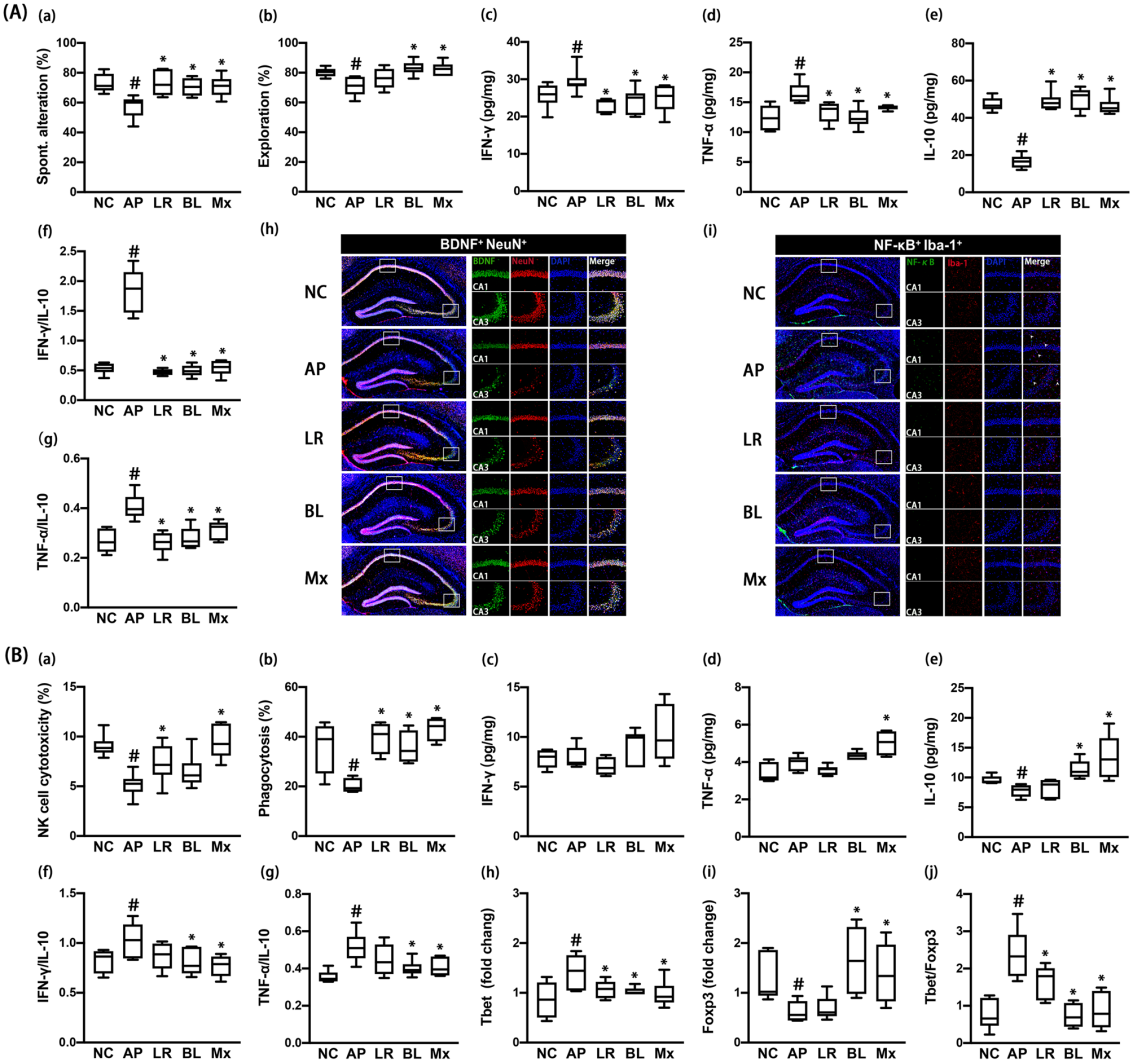
Effects of NK210 and NK219 on ampicillin-induced immune imbalance and cognitive impairment in mice. (**A**) Effects on cognitive impairment in the Y-maze (a) and NOR task (b). Effects on neuroinflammation: IFN-γ (c), TNF-α (d), IL-10 expression (e), IFN-γ to IL-10 expression ratio (f), TNF-α to IL-10 expression ratio (g), and BDNF^+^/NeuN^+^ (h) and NF-κB^+^/Iba1^+^ cell populations (i) in the hippocampus. (**B**) Effects on the immune imbalance: splenic NK cell cytotoxicity activities (a), peritoneal macrophage phagocytosis (b), IFN-γ (c), TNF-α (d), and IL-10 expression (e), IFN-γ to IL-10 expression ratio (f), TNF-α to IL-10 expression ratio (g), Tbet (h) and Foxp3 expression (i), and Tbet to Foxp3 expression ratio (j) in the spleen. Test agents (AP, ampicillin alone; LR, 1 × 10^9^ CFU/mouse/day of NK210; BL, 1 × 10^9^ CFU/mouse/day of NK219; Mx, 1 × 10^9^ CFU/mouse/day of LR and BL [4:1] mix) were orally gavaged daily for 5 days after oral gavage of ampicillin. Normal control mice (NC) were treated with vehicle (saline) instead of test agents. Data values indicate mean ± SD (n = 8). ^#^*p* < 0.05 versus NC group. *p < 0.05 versus AP group treated with ampicillin alone.

Oral gavage of ampicillin causes gut inflammation, gut dysbiosis and depression-like behaviors in mice^25^. First, we examined the effects of NK210 and NK219 on ampicillin-induced cognitive impairment and systemic inflammation in mice. Oral gavage of ampicillin increased cognitive impairment-like behaviors in the Y-maze and NOR tasks (Fig. 3A, Supplementary Figure S3). Ampicillin treatment also increased IFN-γ and TNF-α expression and NF-κB^+^/Iba1^+^ cell population in the hippocampus, while BDNF^+^/NeuN^+^ cell population and IL-10 expression decreased. However, oral administration of NK210, NK219, or Mx decreased cognitive impairment-like behaviors, NF-κB^+^/Iba1^+^ cell population, and IFN-γ and TNF-α expression in the hippocampus, while BDNF^+^/NeuN^+^ cell population and IL-10 expression increased. There also was no the significant difference between NK210, NK219, and Mx. They also suppressed IFN-γ to IL10 and TNF-α to IL-10 expression ratios in the brain.

Oral gavage of ampicillin also decreased the sNK-C and pMA-P activities (Fig. 3B). Furthermore, ampicillin treatment decreased the IL-10 and Foxp3 expression in the spleen, while the Tbet expression increased. The IFN-γ and TNF-α expression was not affected. Nevertheless, ampicillin treatment increased IFN-γ to IL-10, TNF-α to IL-10, and Tbet to Foxp3 expression ratios. However, oral administration of NK210, NK219, or Mx increased peritoneal macrophage phagocytosis. NK210 and Mx, not NK219, significantly increased splenic NK cell cytotoxicity. They all suppressed Tbet to Foxp3 expression ratios in the spleen. NK219 and Mx, not NK210, decreased IFN-γ to IL-10 and TNF-α to IL-10 expression ratios.

**Figure 4:**
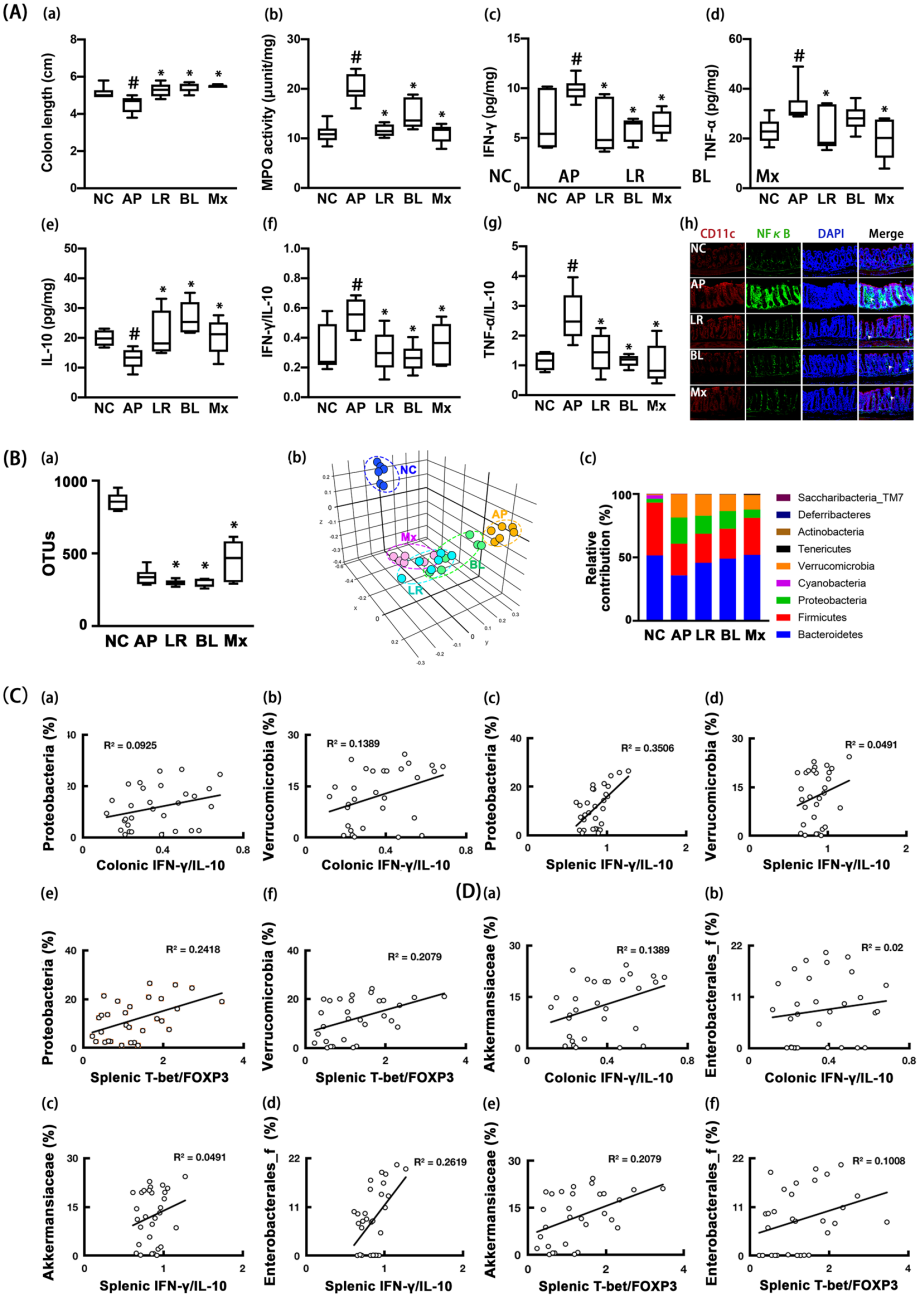
Effects of NK210 and NK219 on ampicillin-induced gut dysbiosis and inflammation in mice. (**A**) Effects on colitis: colon length (a), myeloperoxidase (MPO) activity (b), IFN-γ (c), TNF-α (d), IL-10 expression (e), IFN-γ to IL-10 expression ratio (f), TNF-α to IL-10 expression ratio (g), and NF-κB^+^/CD11c^+^ cell population in the colon (h). (**B**) Effects on the gut microbiota composition: (a) α-diversity (OUT richness, a), (b) β-diversity (PCoA plot based on Jensen-Shannon analysis), and (c) gut microbiota composition at the phylum level. The correlation between gut microbiota and IFN-γ to IL-10 or Tbet to Foxp3 expression ratio at the phylum (**C**) and family levels (**D**). Test agents (AP, ampicillin alone; LR, 1 × 10^9^ CFU/mouse/day of NK210; BL, 1 × 10^9^ CFU/mouse/day of NK219; Mx, 1 × 10^9^ CFU/mouse/day of LR and BL [4:1] mix) were orally gavaged daily for 5 days after oral gavage of ampicillin. Normal control mice (NC) were treated with vehicle (saline) instead of test agents. Data values indicate mean ± SD (n = 8). ^#^*p* < 0.05 versus NC group. *p < 0.05 versus AP group treated with ampicillin alone.

Moreover, oral gavage of ampicillin induced colitis: it increased colon shortening, myeloperoxidase activity, and IFN-γ and TNF-α expression and reduced IL-10 expression in the colon (Fig. 4A). Furthermore, ampicillin treatment increased IFN-γ to IL-10 and TNF-α to IL-10 expression ratios. However, oral administration of NK210, NK219, or Mx alleviated ampicillin-induced colitis. They decreased colon shortening, IFN-γ expression, and NF-κB^+^/CD11c^+^ cell population in the colon and increased IL-10 expression. They reduced IFN-γ to IL-10 and TNF-α to IL-10 expression ratios.

Next, we examined the effects of NK210 and NK219 on ampicillin-induced gut dysbiosis in mice. Oral gavage of ampicillin significantly caused gut dysbiosis: it suppressed the α-diversity (OTUs) and shifted the β-diversity (PCoA) (Fig. 4B, Supplementary Tables S1–S4). Ampicillin treatment also altered the gut microbiota composition: they suppressed the Bacteroidetes and Firmicutes populations at the phylum level, while Proteobacteria and Verrucomicrobita populations were increased. Oral administration of NK210, NK219, or Mx partially restored the gut microbiota composition to that of normal control mice. They shifted the α- and β-diversities of ampicillin-induced gut microbiota to those of normal control mice. Of these, Mx most potently shifted them. They also increased Firmicutes and Bacteroidetes populations including Lachnosipracease, Muribaculaceae, and Prevotellaceae and decreased Proteobacteria and Verrucomicrobia populations including Bacteroidaseceae, Erysipelotrichaceae, Sutterellaceae, and Enterobacterales_f in the gut microbiota of mice with LPS-induced systemic inflammation. The correlation between gut microbiota populations and IFN-γ to IL-10 or Tbet to Foxp3 expression ratio was also analyzed in the gut and spleen (Fig. 4C,D). At the phylum level, Verrucomicrobia (R = 0.37 in colon, *p* = 0.043 in the colon) and Proteobacteria populations (R = 0.59, *p* = 0.001 in spleen) was positively correlated with the IFN-γ to IL-10 expression ratio, while Bacteroidetes (R = − 0.47, *p* = 0.010 in the spleen) population was negatively correlated with it. Firmicutes population was negatively correlated with the splenic Tbet to Foxp3 expression ratio, while Verrucomicrobia (R = 0.46, *p* = 0.011) and Proteobacteria populations (R = 0.49, *p* = 0.001) were positively correlated with it. At the family level, Lachnospiraceae, Prevotellaceae, and Helicobacteriaceae populations were negatively correlated with IFN-γ to IL-10 and Tbet to Foxp3 expression ratios, while Erysipeltrichaceae, Akkermaniaceae, and Bacteroidaeceae populations were positively correlated with them.

### Effects of NK210 and NK219 on cognitive impairment and colitis in mice with cyclophosphamide-induced immunosuppression

**Figure 5:**
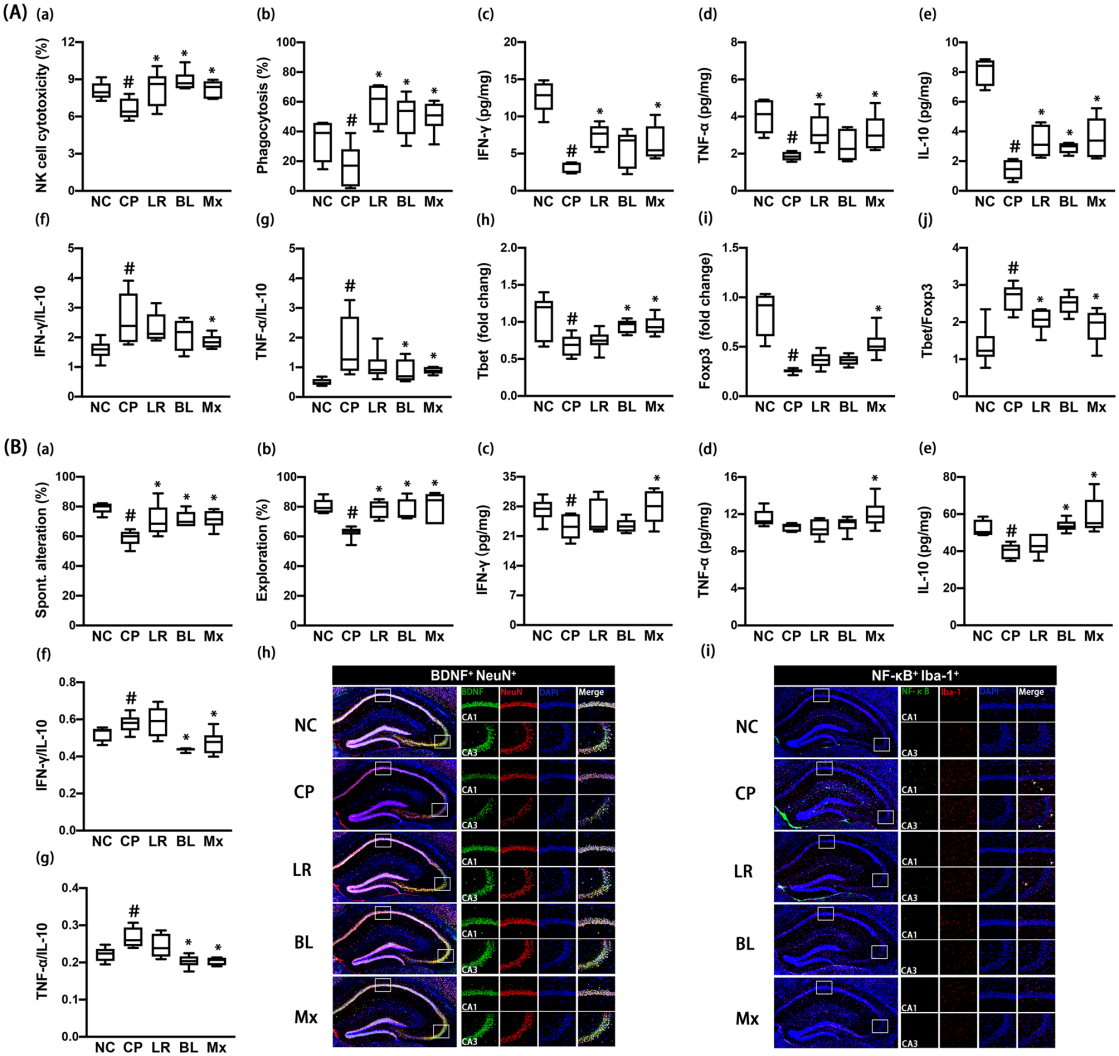
Effects of NK210 and NK219 on cyclophosphamide-induced immune imbalance and cognitive impairment in mice. (**A**) Effects on the immune imbalance: splenic NK cell cytotoxicity activities (a), peritoneal macrophage phagocytosis (b), IFN-γ (c), TNF-α (d), and IL-10 expression (e), IFN-γ to IL-10 expression ratio (f), TNF-α to IL-10 expression ratio (g), Tbet (h) and Foxp3 expression (i), and Tbet to Foxp3 expression ratio (j) in the spleen. (**B**) Effects on cognitive impairment in the Y-maze (a) and NOR task (b). Effects on neuroinflammation: IFN-γ (c), TNF-α (d), IL-10 expression (e), IFN-γ to IL-10 expression ratio (f), TNF-α to IL-10 expression ratio (g), and BDNF^+^/NeuN^+^ (h) and NF-κB^+^/Iba1^+^ cell populations (i) in the hippocampus. Test agents (CP, cyclophosphamide alone; LR, 1 × 10^9^ CFU/mouse/day of NK210; BL, 1 × 10^9^ CFU/mouse/day of NK219; Mx, 1 × 10^9^ CFU/mouse/day of LR and BL [4:1] mix) were orally gavaged daily for 5 days after intraperitoneal injection of cyclophosphamide. Normal control mice (NC) were treated with vehicle (saline) instead of test agents. Data values indicate mean ± SD (n = 8). ^#^*p* < 0.05 versus NC group. *p < 0.05 versus CP group treated with cyclophosphamide alone.

Cyclophosphamide, an antitumor drug, suppresses the immune response in mice^26^. To understand the effects of gut lactobacilli and bifidobacteria in immune-suppressed hosts, we examined the effects of NK210, NK219, and Mx on the immune homeostasis in mice with cyclophosphamide-induced immunosuppression. Intraperitoneal injection of cyclophosphamide suppressed the sNK-C and pMA-P activities (Fig. 5A). Furthermore, cyclophosphamide treatment suppressed IFN-γ, TNF-α, IL-10, Tbet, and Foxp3 expression in the spleen. However, it increased IFN-γ to IL-10, TNF-α to IL-10, and Tbet to Foxp3 expression ratios. However, oral administration of NK210, NK219, or Mx restored cyclophosphamide-suppressed splenic sNK-C and pMA-P activities. They increased IL-10 expression in the spleen. NK210 and Mx, not NK219, significantly increased cyclophosphamide-suppressed IFN-γ and TNF-α expression. NK210 and NK219 weakly, not significantly, suppressed IFN-γ to IL-10, TNF-α to IL-10, and Tbet to Foxp3 expression ratios. However, Mx significantly suppressed them.

Cyclophosphamide treatment also increased cognitive impairment-like behaviors in the Y-maze and NOR tasks (Fig. 5B, Supplementary Figure S4). It decreased IFN-γ and IL-10 expression and BDNF^+^/NeuN^+^ cell population in the hippocampus, while NF-κB^+^/Iba1^+^ cell population increased. However, TNF-α expression was not affected by its treatment. Nevertheless, cyclophosphamide treatment increased IFN-γ to IL-10 and TNF-α to IL-10 expression ratios in the hippocampus. However, oral administration of NK210 and/or NK219 decreased cognitive impairment-like behaviors and NF-κB^+^/Iba1^+^ cell population in the hippocampus, while BDNF^+^/NeuN^+^ cell population increased. NK219 and Mx, not NK210, increased IL-10 expression. Mx only increased TNF-α and IFN-γ expression. NK219 and Mx, not NK210, suppressed the IFN-γ to IL-10 and TNF-α to IL-10 expression ratios in the hippocampus.

**Figure 6:**
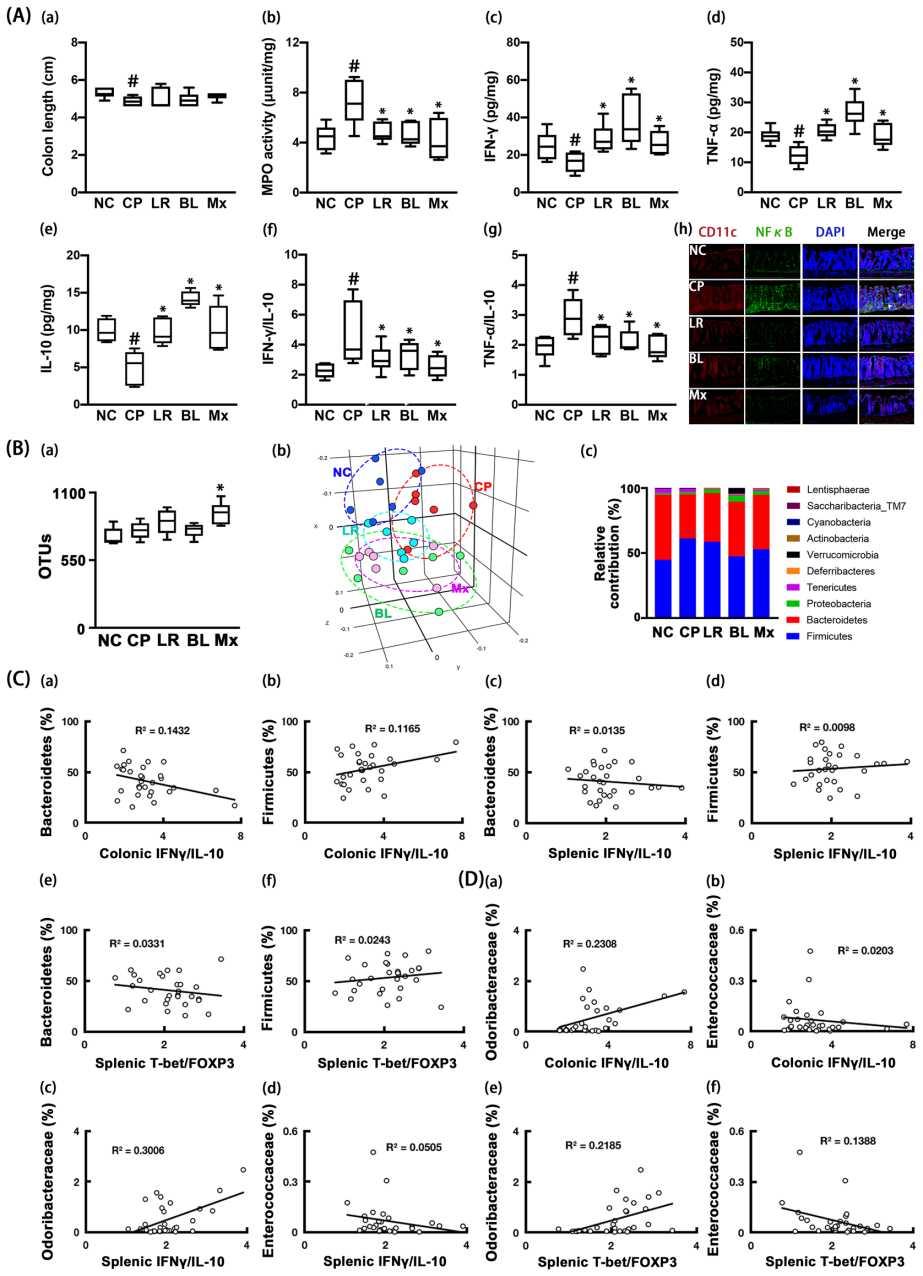
Effects of NK210 and NK219 on cyclophosphamide-induced gut inflammation and microbiota alteration in mice. (**A**) Effects on colitis: colon length (a), myeloperoxidase (MPO) activity (b), IFN-γ (c), TNF-α (d), IL-10 expression (e), IFN-γ to IL-10 expression ratio (f), TNF-α to IL-10 expression ratio (g), and NF-κB^+^/CD11c^+^ cell population in the colon (h). (**B**) Effects on the gut microbiota composition: (a) α-diversity (OUT richness, a), (b) β-diversity (PCoA plot based on Jensen-Shannon analysis), and (c) gut microbiota composition at the phylum level. The correlation between gut microbiota and IFN-γ to IL-10 or Tbet to Foxp3 expression ratio at the phylum (**C**) and family levels (**D**). Test agents (CP, cyclophosphamide alone; LR, 1 × 10^9^ CFU/mouse/day of NK210; BL, 1 × 10^9^ CFU/mouse/day of NK219; Mx, 1 × 10^9^ CFU/mouse/day of LR and BL [4:1] mix) were orally gavaged daily for 5 days after intraperitoneal injection of cyclophosphamide. Normal control mice (NC) were treated with vehicle (saline) instead of test agents. Data values indicate mean ± SD (n = 8). ^#^*p* < 0.05 versus NC group. *p < 0.05 versus CP group treated with cyclophosphamide alone.

Intraperitoneal injection of cyclophosphamide caused colon shortening and increased myeloperoxidase activity and NF-κB^+^/CD11c^+^ cell population in the colon, while IFN-γ, TNF-α, and IL-10 expression decreased (Fig. 6A). Nevertheless, cyclophosphamide treatment increased IFN-γ to IL-10 and TNF-α to IL-10 expression ratios. However, oral gavage of NK210 and/or NK219 suppressed cyclophosphamide-induced colitis: they increased IFN-γ, TNF-α, and IL-10 expression, but decreased myeloperoxidase activity and IFN-γ to IL-10 and TNF-α to IL-10 expression ratios in the colon.

Next, we examined the effects of NK210 and/or NK219 on the gut microbiota composition in mice with cyclophosphamide-induced immunosuppression. Oral gavage of cyclophosphamide weakly caused gut dysbiosis: it increased the α-diversity and shifted the β-diversity (PCoA) (Fig. 6B, Supplement Tables S5–S8). Cyclophosphamide treatment altered the gut microbiota composition: it decreased Bacteroidetes population at the phylum level, while Firmicutes population increased. However, oral administration of NK210, NK219, or Mx weakly shifted the α- and β-diversities of cyclophosphamide-induced gut microbiota. They increased cyclophosphamide-suppressed Bacteroidetes population including Muribaculaceae, Porphyromonadaceae, and Sutterellaceae and decreased cyclophosphamide-induced Firmicutes population including Odoribacteriaceae. The correlation between gut microbiota populations and IFN-γ to IL-10 or Tbet to Foxp3 expression ratio was also analyzed in the gut and spleen (Fig. 6C,D). At the phylum level, Bacteroidetes population (R = − 0.38, *p* = 0.039 in the colon) was positively correlated with the IFN-γ to IL-10 expression ratio, while Firmicutes and Proteobacteria populations were negatively correlated with it. Proteobacteria and Firmicutes populations were positively correlated with the Tbet to Foxp3 expression ratio, while Bacteroidetes population was negatively correlated with it. At the family level, Bacteroidaceae, Enterococcaceae, and Rikenellaceae populations were negatively correlated with IFN-γ to IL-10 and Tbet to Foxp3 expression ratios, while Odoribacteaceae and Desulfovibrionaceae populations were positively correlated with them.

### NK219 potentiated colonic IFN-γ to IL-10 expression ratios in normal control mice

**Figure 7:**
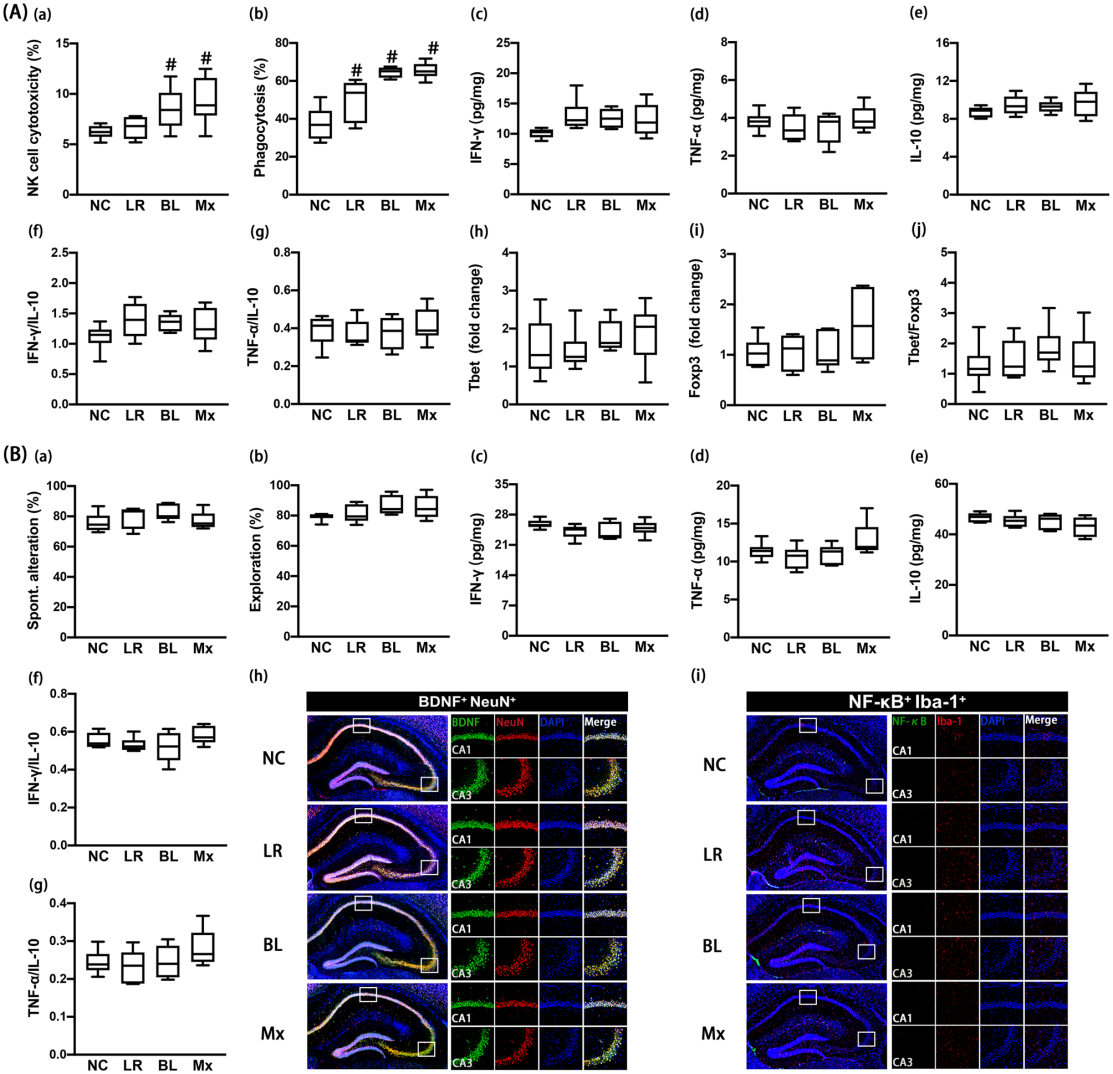
Effects of NK210 and NK219 on systemic immune response and cognitive function in mice. (**A**) Effects on the immune imbalance: splenic NK cell cytotoxicity activities (a), peritoneal macrophage phagocytosis (b), IFN-γ (c), TNF-α (d), and IL-10 expression (e), IFN-γ to IL-10 expression ratio (f), TNF-α to IL-10 expression ratio (g), Tbet (h) and Foxp3 expression (i), and Tbet to Foxp3 expression ratio (j) in the spleen. (**B**) Effects on cognitive impairment in the Y-maze (a) and NOR task (b). Effects on neuroinflammation: IFN-γ (c), TNF-α (d), IL-10 expression (e), IFN-γ to IL-10 expression ratio (f), TNF-α to IL-10 expression ratio (g), and BDNF^+^/NeuN^+^ (h) and NF-κB^+^/Iab1^+^ cell populations (i) in the hippocampus. NK210 (LR, 1 × 10^9^ CFU/mouse/day), NK210 (BL, 1 × 10^9^ CFU/mouse/day) or their mix (Mx, 1 × 10^9^ CFU/mouse/day of LR and BL [4:1] mix) were orally gavaged daily for 5 days. Normal control mice (NC) were treated with vehicle (saline) instead of test agents. Data values indicate mean ± SD (n = 8). ^#^*p* < 0.05 versus NC group.

We examined the effect of NK210 and NK219 on the immune response in normal control mice. Oral administration of NK219, NK210, and Mx increased the sNK-C activities. Treatments with NK219 and Mx, not NK210, increased the pMA-P activities (Fig. 7A). However, they did not influence of IFN-γ, TNF-α, IL-10, Tbet, and Foxp3 expression in the spleen. Moreover, they did not affect the cognitive function-like behaviors and BDNF^+^/NeuN^+^ and NF-κB^+^/Iba1^+^ cell populations and IFN-γ, TNF-α, and IL-10 expression in the hippocampus (Fig. 7B, Supplementary Figure S5).

**Figure 8:**
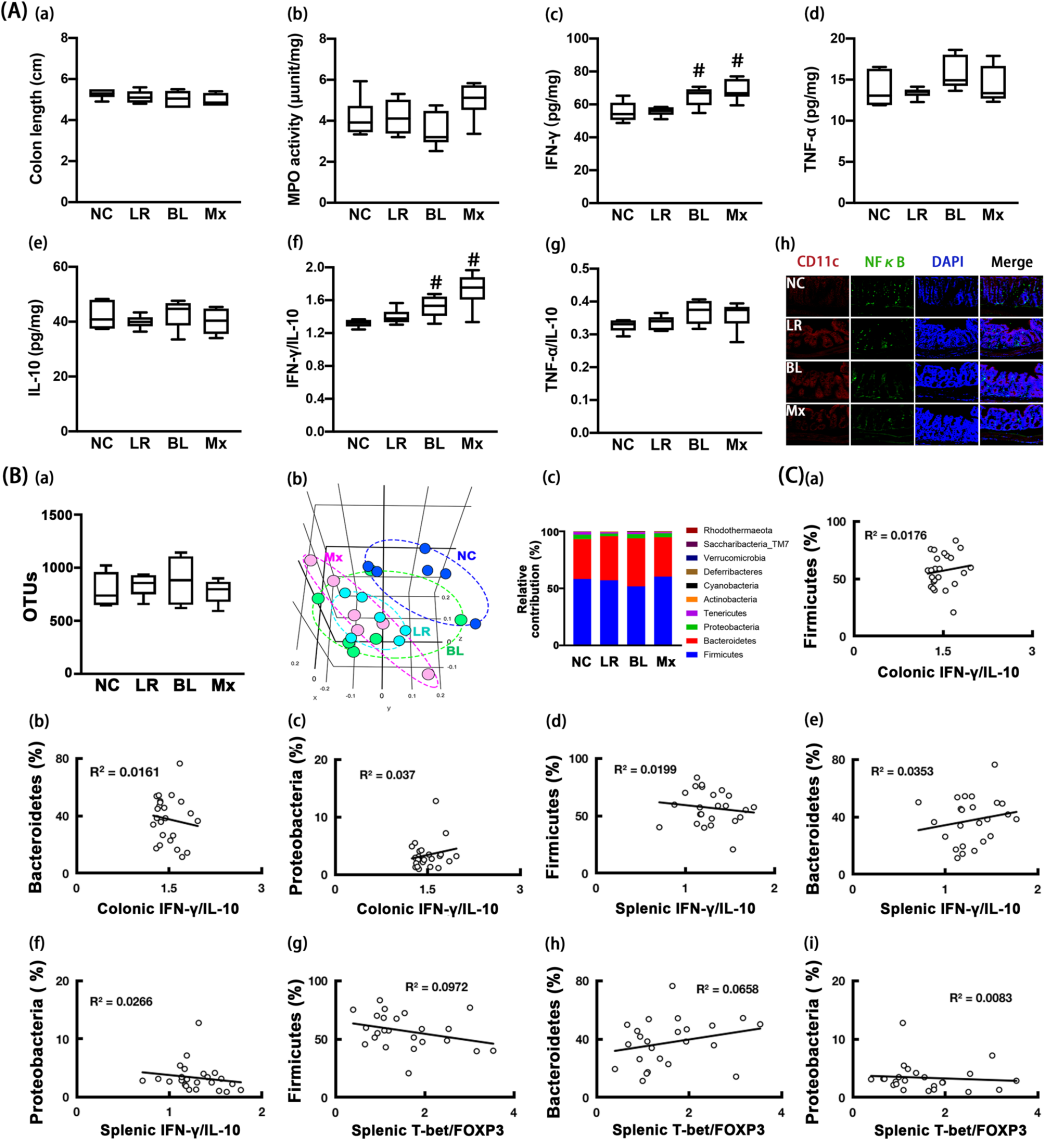
Effects of NK210 and NK219 on gut immune response and microbiota composition in mice. (**A**) Effects on colitis: colon length (a), myeloperoxidase (MPO) activity (b), IFN-γ (c), TNF-α (d), IL-10 expression (e), IFN-γ to IL-10 expression ratio (f), TNF-α to IL-10 expression ratio (g), and NF-κB^+^/CD11c^+^ cell population in the colon (h). (**B**) Effects on the gut microbiota composition: (a) α-diversity (OUT richness, a), (b) β-diversity (PCoA plot based on Jensen-Shannon analysis), and (c) gut microbiota composition at the phylum level. The correlation between gut microbiota and IFN-γ to IL-10 or Tbet to Foxp3 expression ratio at the phylum (**C**) and family levels (**D**). NK210 (LR, 1 × 10^9^ CFU/mouse/day), NK210 (BL, 1 × 10^9^ CFU/mouse/day) or their mix (Mx, 1 × 10^9^ CFU/mouse/day of LR and BL [4:1] mix) were orally gavaged daily for 5 days. Normal control mice (NC) were treated with vehicle (saline) instead of test agents. Data values indicate mean ± SD (n = 8). ^#^*p* < 0.05 versus NC group.

Oral administration of NK210 and/or NK219 did not affect colitis-related colon shortening, myeloperoxidase activity, and TNF-α and IL-10 expression in the colon (Fig. 8A). However, NK219 and Mx, not NK210, significantly increased IFN-γ expression, resulting to the increase in the IFN-γ to IL-10 expression ratio. Moreover, oral gavage of NK210, NK219, or Mx weakly shifted the the β-diversity (PCoA), while the α-diversity was not affected (Fig. 8B, Supplementary Tables S9–S12). Their treatment did not significantly influence the gut microbiota composition at the phylum level. They suppressed the Rikenellaceae and Helicobacteriaceae populations at the family level, Alistipes and PAC00128_g and HM630235_g populations at the genus level, and PAC002444_s, PAC001070_s group, PAC000198_s, PAC001060_s group populations at the species level, while the PAC000186_g population increased. When the correlation between gut microbiota populations and IFN-γ to IL-10 or Tbet to Foxp3 expression ratio was analyzed in the gut and spleen, Ternericutes population (R = 0.51, *p* = 0.011) was positively correlated with the Tbet to Foxp3 expression ratio at the phylum level, Firmicutes, Protoebacteria, and Bacteroidetes populations were not correlated with the IFN-γ to IL-10 or Tbet to Foxp3 expression ratio (Fig. 8C).

## Discussion

The immune system has two fundamental lines of defense against pathogens and their toxins: innate immunity and adaptive immunity^27,28^. The attack of pathogenic microbes and toxins such as *Escherichia coli* and LPS activate innate immune cells, such as dendritic cells, macrophages, and NK cells, which secrete IFN-γ, TNF-α, IL-1β, and IL-10^29,30^. These cytokines activate innate and adaptive immune cells such as T cells and differentiate naïve T cells into Th1, Th2, Th17, and Treg cells^28,30^. Chronic exposure to pathogens and their toxins can cause chronic inflammation through the activation of antigen-presenting cells, Th1, and Th17 cells and suppression of Treg cells^30,31^. IFN-γ and TNF-α, inflammatory cytokines, activate macrophages, dendritic, Th1, and Th17 cells while Treg cells are suppressed. IL-10, an anti-inflammatory cytokine, activates Treg cells but suppresses activate macrophages, dendritic, Th1, and Th17 cells^28,31^. However, cyclophosphamide, an anticancer drug, suppresses the immune systems including macrophages and T cells and down-regulates the immune defense against pathogens and their toxins^26,32^. These immune imbalances perturb the gut microbiota composition by regulating the expression of these cytokines such as IFN-γ, TNF-α, and IL-10. Therefore, the physiological effects of gut bacteria including bifidobacteria and lactobacilli may be dependent on the regulation of the expression of inflammatory and anti-inflammatory cytokines.

In the present study, we found that exposure to LPS, ampicillin, or cyclophosphamide caused colitis, gut dysbiosis, and cognitive impairment in mice. However, they significantly differently regulated the expression of immune cytokines and T cell transcription factors. Exposure to LPS or ampicillin increased the expression of IFN-γ, TNF-α, and Tbet expression, while IL-10 and Foxp3 expression decreased. However, cyclophosphamide decreased the IFN-γ, TNF-α, IL-10, Tbet, and Foxp3 expression. Moreover, LPS treatment increased sNK-C and pMA-P activities, while treatment with ampicillin or cyclophosphamide decreased sNK-C and pMA-P activities. However, LPS, ampicillin, and cyclophosphamide treatments increased NF-κB^+^/CD11c^+^ cell population in the colon and NF-κB^+^/Iba1^+^ cell population in the hippocampus. Moreover, they all increased the IFN-γ to IL-10, TNF-α to IL-10, and Tbet to Foxp3 expression ratios. IFN-γ, TNF-α, and IL-10 are expressed in innate and adaptive immune cells such as macrophages and T cells^33,34^. IFN-γ and TNF-α induce the expression of Tbet expression, a Th1 cell transcription factor, and IL-10 induces the expression of Foxp3, a Treg cell transcription factor. Tbet and Foxp3 stimulate Th1 and Treg cell differentiation, respectively^35,36^. Th1 cells control IFN-γ and TNF-α expression, while Treg cells control IL-10 expression. Jang et al. reported that ampicillin treatment significantly caused colitis, gut dysbiosis, and anxiety in mice and increased TNF-α expression in the colon and hippocampus^25^. Lee et al. reported that LPS treatment caused cognitive impairment, colitis and gut dysbiosis and increased TNF-α expression in the colon and hippocampus^24^. Jang et al. reported that cyclophosphamide treatment suppressed sNK-C and pMA-P activities, T cell differentiation, and IFN-γ expression in mice^26^. These results suggest that stressors such as LPS, ampicillin, and cyclophosphamide can cause the hypersensitivity (inflammation) and/or immunosuppression by regulating the IFN-γ to IL-10 and TNF-α to IL-10 expression ratios in the immune cells.

In the present study, NK210 and/or NK219, which were isolated from human feces, significantly suppressed LPS- or ampicillin-induced TNF-α, IFN-γ, and Tbet expression and NF-κB^+^/Iba1^+^ cell population in mice, while LPS- or ampicillin-suppressed IL-10 and Foxp3 expression and BDNF^+^/NeuN^+^ cell population increased. However, they increased cyclophosphamide-suppressed IFN-γ, TNF-α, IL-10, Tbet, and Foxp3 expression in the colon, spleen, and hippocampus. Interestingly, they suppressed the LPS-, ampicillin- and cyclophosphamide-induced TNF-α to IL-10 and IFN-γ to IL-10 expression ratios in the colon, spleen, and hippocampus. Moreover, they suppressed LPS-induced TNF-α to IL-10 and IFN-γ to IL-10 expression ratios in macrophages in vitro (Fig. 1, Supplementary Figure S6). They also suppressed LPS-induced sNK-C and pMA-P activities, while ampicillin- or cyclophosphamide-suppressed sNK-C and pMA-P activities were induced. Furthermore, NK210 and/or NK219 alleviated LPS-, ampicillin- and cyclophosphamide-induced colitis and cognitive impairment in mice. Jang et al. reported that *Lactobacillus johnsonii* alleviated ampicillin-induced colitis, gut dysbiosis, and depression, and TNF-α expression in the colon and hippocampus of mice^25^. Lee et al. reported that *Lactobacillus plantarum* C29 alleviated LPS-induced colitis, gut dysbiosis, and cognitive impairment and TNF-α expression in mice by regulating NF-κB activation-mediated BDNF expression^24^. Jang et al. reported that *Lactobacillus casei* HY7213 alleviated cyclophosphamide-induced suppression of NK and Tc cell cytotoxicity and macrophage phagocytosis in mice by enhancing IL-2 and IFN-γ expression^26^. These results suggest that NK210 and NK219 can alleviate colitis and cognitive impairment by regulating the expression of inflammatory and anti-inflammatory cytokines (IFN-γ to IL-10 and/or TNF-α to IL-10 expression ratios).

NK210 and NK219 did not affect cognitive function and colitis in healthy control mice. NK210 treatment did not significantly affect TNF-α, IL-10, Tbet, and Foxp3 expression in the colon and spleen. However, oral administration of NK219, not NK210, significantly increased the sNK-C and p-MA-P activities. NK219, not NK210, induced IFN-γ expression and IFN-γ to IL-10 expression ratio in the colon. Furthermore, NK219 increased IFN-γ to IL-10 expression ratio in the resident macrophages. *Lactobacilli* including *Lactobacillus rhamnosus* HDB1258 and *Lactobacillus casei* CRL431 enhance the innate immunity including to macrophage phagocytosis and NK cell cytotoxicity in healthy hosts by increasing TNF-α and IFN-γ expression in immune cells^37^. Bifidobacteria including *Bifidobacterium longum* NCC2705 activate innate and adaptive immune cells by increasing IFN-γ expression^38,39^. These results suggest that the induction of IFN-γ expression by gut microbiota can activate innate immune cells including macrophages and NK cells in the healthy host. The cotreatment with NK219 and NK210 additively or synergistically increased sNK-C and p-MA-P activities, and IFN-γ to IL-10 expression ratio, like treatment with NK219 alone. However, NK219, not NK210, induced IFN-γ expression in resident macrophages. Nevertheless, they did not antagonistically regulate the immune homeostasis in vitro and in vivo. These results suggest that gut bacteria can cooperatively modulate the immune response by regulating IFN-γ to IL-10 expression ratio.

Ampicillin and cyclophosphamide shift gut microbiota composition in mice, as previously reported^25,40^. Ampicillin treatment significantly increased Proteobacteria and Verrucomicrobiota populations, which were positively correlated with the IFN-γ to IL-10 and Tbet to Foxp3 expression ratios. Cyclophosphamide treatment decreased the Bacteroidetes population and increased the Firmicutes population. The former were negatively correlated with IFN-γ to IL-10 or Tbet to Foxp3 expression ratio, while the letter were positively with them. Treatment with NK210 and/or NK219 alleviated the immune imbalance as well as gut microbiota alteration. However, treatment of healthy control mice with NK210 or NK219 did not significantly affect gut microbiota composition, while treatment with NK219, not NK210, induced macrophage phagocytosis and NK cell cytotoxicity activities in vivo, IFN-γ expression, and IFN-γ to IL-10 expression ratio in the colon. These results suggest that gut microbiota such as NK219 and NK210 can cooperatively modulate gut microbiota composition as well as gut immunity in vivo.

In conclusion, NK210, Lactobacillus sp, and NK219, Bifidobacterium additively or synergistically control IFN-γ to IL-10 and TNF-α to IL-10 expression ratios in the host with and without immune imbalance, resulting in the alleviation of gut dysbiosis, inflammation, and cognitive impairment.

## Materials and methods

### Materials

Sodium thioglycolate, 4′,6-diamidino-2-phenylindole dilactate (DAPI), LPS, and RPMI 1640 were purchased from Sigma (St Louis, MO). A De Man, Rogosa, and Sharpe (MRS) medium, Sabouraud dextrose agar (SDA), and general anaerobic medium were purchased from BD (Franklin Lakes, NJ). An antibody for NF-κB was purchased from Cell Signaling Technology (Danvers, MA). Antibodies for CD11c, Iba1, and BDNF were purchased from Abcam (Cambridge, U.K.). Alexa Fluor 488 and Alexa Fluor 594 were purchased from Invitrogen (Carbsband, CA). Enzyme-linked immunosorbent assay (ELISA) kits for IFN-γ, TNF-α, and IL-10 were purchased from Ebioscience (Atlanta, GA). A QIAamp Fast DNA stool mini kit was purchased from Qiagen (Hilden, Germany). EasyTaq DNA polymerase and 100 bp plus II DNA ladder were purchased from TransGen Biotech (Beijing, China). TB Green® Premix Ex Taq™ II was purchased from Takara Bio (Shiga, Japan).

### Culture of gut bacteria and Candida albicans

Bifidibacteria and lactobacilli including NK210 and NK219 were selected from human fecal bacteria collection previously prepared in Neurobiota Research Center (Kyung Hee University, Seoul, Korea), cultured in commercial media for probiotics including MRS broth, centrifuged (5000 g, 4 °C, 20 min), and washed with saline, as previously reported^41^. The collected cells were used for further experiments.

*Candida albicans* (purchased from Korean Culture Center of Microorganisms, Seoul, Korea) was cultured in SDA agar plates, collected in sterilized test tube, and washed with saline, and suspended in saline for macrophage phagocytic activity assay.

### Animals

SPF C57BL/6 male mice (19–22 g, 6 weeks old) were purchased from Koatech (Pyungtaek-shi, Korea). All mice were maintained in plastic cages with the raised wire floor at 20–22 °C and 50 ± 10% humidity, and fed standard laboratory chow and water ad libitum. Each group consisted of 8 mice (4 mice in each cage). The animals were acclimatized for one week before usage in the experiments. All animal experiments were approved by The Committee for the Care and Use of Laboratory Animals in Kyung Hee University (Seoul, Korea) and performed in the accordance to The Kyung Hee University Guidelines for Laboratory Animals Care and Usage (IACUC No., KHSASP-20018). This study additionally adheres to standards articulated in the ARRIVE guidelines.

### Culture of macrophages

Macrophages were removed from the peritoneal cavity of SPF C57BL/6 male mice injected intraperitoneally with sodium thioglycolate, suspended in RPMI 1640 containing 10% fetal bovine serum and 1% antibiotics (RFA), seeded on a 12-well plate, incubated at 37 °C for 6 h, and washed with RFA, as reported previously^18^. Macrophage cells were then incubated with LPS (100 ng/mL) in the absence or presence of NK210 or NK219 (1 × 10^4^ or 1 × 10^6^ CFUs/mL) for 20 h. IFN-γ and IL-10 levels were assayed.

### Preparation of mice with systemic inflammation, gut dysbiosis, or immune suppression

For the assay of the immunomodulating activity in vivo, we prepared mice with LPS-induced systemic inflammation, ampicillin-induced gut dysbiosis, and cyclophosphamide-induced immunosuppression (Supplementary Figure S7), as reported previously^24–26^.

First, mice with systemic inflammation were prepared by intraperitoneally injecting LPS (10 μg/kg, dissolved in 0.1 mL of saline) once a day for 5 days. Probiotics (LR, 1 × 10^9^ CFU/mouse/day of NK210; BL, 1 × 10^9^ CFU/mouse/day of NK219; and Mx, 1 × 10^9^ CFU/mouse/day of NK210 and NK219 [4:1] mixture) were orally gavaged in mice with LPS-induced systemic inflammation once a day for 5 days from 24 h after the final injection of LPS. Control group was orally gavaged with vehicle (saline) instead of probiotics. Each group consisted of 8 mice (4 mice in each cage).

Second, mice with gut dysbiosis were prepared by orally gavaging ampicillin (100 mg/kg, dissolved in 0.1 mL of saline) once a day for 3 days. Probiotics (LR, 1 × 10^9^ CFU/mouse/day of NK210; BL, 1 × 10^9^ CFU/mouse/day of NK219; and Mx, 1 × 10^9^ CFU/mouse/day of NK210 and NK219 [4:1] mixture) were orally gavaged in mice with ampicillin-induced gut dysbiosis once a day for 5 days from 24 h after the final gavage of ampicillin. Control group was orally gavaged with vehicle (saline) instead of probiotics.

Third, mice with immunosuppression were prepared by intraperitoneally injecting with cyclophosphamide (cyclophosphamide, 150 mg/kg/day) twice (the 1st and 3rd day) according to Jang et al.^26^ Probiotics (LR, 1 × 10^9^ CFU/mouse/day of NK210; BL, 1 × 10^9^ CFU/mouse/day of NK219; and Mx, 1 × 10^9^ CFU/mouse/day of NK210 and NK219 [4:1] mixture) were orally gavaged in mice with cyclophosphamide-induced immunosuppression once a day for 5 days from 24 h after the final injection of cyclophosphamide. Control group was orally gavaged with vehicle (saline) instead of probiotics.

Fourth, probiotics (LR, 1 × 10^9^ CFU/mouse/day of NK210; BL, 1 × 10^9^ CFU/mouse/day of NK219; and Mx, 1 × 10^9^ CFU/mouse/day of NK210 and NK219 [4:1] mixture) were orally gavaged in healthy mice once a day for 5 days. Control group was orally gavaged with vehicle (saline) instead of probiotics.

Cognitive function-like behaviors in the Y-maze and novel object recognition (NOR) tasks were performed 20 h after the final treatment of probiotics according to the the method of Jeong et al.^42^ Eighteen hours after the final behavioral task, mice were euthanized by the inhalation of CO^2^. Colons, brains, spleens, and stools were removed after the final treatment with test agents by CO_2_ inhalation and stored at − 80 °C for further experiments.

### Phagocytosis assay of peritoneal macrophages

Macrophages (1 × 10^6^ cell/well) were incubated with *Candida albicans* (1 × 10^4^ CFU/well) in the 24-well microplates with RPMI 1640 medium for 24 h. Cultured supernatant (0.2 mL) was cultured in SDA plates for 24 h at 30°C^43^. The phagocytic activity (%) was indicated as [1-(the number of *Candida albicans* colonies grown in SDA per the number of *Candida albicans* initially incubated with macrophages)] × 100.

### Cytotoxicity assay of NK cells

NK cells were isolated from splenocytes prepared from the spleen of mice using a NK cell isolation kit and suspended in RFA, as described previously^43^. NK cell cytotoxicity was assayed by measuring the cytotoxicity against YAC-1 cells labeled with a Vybrant CFDA SE Cell Tracer kit according to the manufacturer’s protocol. Briefly, NK cells (5 × 10^5^ per well) in the 96-well microplates were cultured with YAC-1 cells (3 × 10^5^ per well) for 24 h, washed with RFA, and stained with propodium iodide, as reported previously. Stained cells were counted by a flow cytometer.

### ELISA and myeloperoxidase activity assay

For the assay of cytokines, colon homogenate supernatants were transferred in 96-well plate and assayed using ELISA Kits (Ebioscience, San Diego, CA). Myeloperoxidase activity was assayed according to the method of Kim et al.^15^.

### Quantitative real time-polymerase chain reaction (qPCR)

qPCRs were carried out according to the method of Kim et al.^15^ Briefly, mRNA (2 μg) was isolated from the spleen of mice. qPCRs for Tbet, Foxp3, and glyceraldehyde 3-phosphate dehydrogenase (GAPDH) were performed utilizing Takara thermal cycler, which used SYBER premix agents: activation of DNA polymerase at 95 °C for 5 min and 45 cycles of amplification at 95 °C for 10 s and at 60 °C for 30 s^15^. The normalized expression of Foxp3, Tbet, and GAPDH genes (their primers are described in Table S13), with respect to GAPDH, was calculated.

### Immunofluorescence assay

Immunofluorescence assay was performed according to the method of Kim et al.^15^ Briefly, the colon section was washed with phosphate-buffered saline, incubated with an antibody for BDNF, NeuN, NF-κB, CD11c, or Iba1 overnight, and treated with the secondary antibody for 2 h. A secondary antibody conjugated with Alexa Fluor 488 or Alexa Fluor 594 was incubated to visualize. Nuclei were stained with DAPI. The stained sections were observed using a confocal microscope.

### Gut microbiota composition assay

The bacterial genomic DNA was extracted for the fresh feces of mice using a QIAamp DNA stool mini kit according to Kim et al.^15^ The genomic DNA was amplified using barcoded primers targeted the bacterial 16S rRNA V4 region gene. Each amplicon was sequenced using Illumina iSeq 100 (San Diego, CA). Functional genes was predicted using the phylogenetic investigation of communities by reconstruction of unobserved states^15,44,45^. Data availability 16S sequencing dataset (pyrosequencing reads) was deposited in the NCBI’s short read archive under accession number PRJNA741596.

### Statistical analysis

All data are indicated as the means ± standard deviation (SD) and conducted GraphPad Prism 9 (GraphPad Software, Inc., San Diego, CA, USA). The significance was analyzed using one-way ANOVA, followed by Dunnett’s multiple comparisons, or Kruskal–Wallis test (*p* < 0.05).

## Supplementary Information

Supplementary Information.
