# Author Correction: *Lactobacillus rhamnosus* and *Bifidobacterium longum* alleviate colitis and cognitive impairment in mice by regulating IFN-γ to IL-10 and TNF-α to IL-10 expression ratios

**DOI:** 10.1038/s41598-023-27871-2

**Published:** 2023-01-13

**Authors:** Xiaoyang Ma, Yoon-Jung Shin, Hyo-Min Jang, Min-Kyung Joo, Jong-Wook Yoo, Dong-Hyun Kim

**Affiliations:** Neurobiota Research Center, College of Pharmacy, Kyung Hee University, 26, Kyungheedae-Ro, Dongdaemun-Gu, Seoul, 02447 Korea

Correction to: *Scientific Reports* 10.1038/s41598-021-00096-x, published online 19 October 2021

The original version of this Article contained an error in the legend of Figure 1.

“Effect of NK210 and NK219 on the expression of interferon (IFN)-γ and IL-10 in macrophages stimulated with or without LPS. (**A**) Effect on IFN-γ (a) and IL-10 expression (b) and IFN-γ to IL-10 expression ratio (c) in LPS-stimulated macrophage cells. (**B**) Effect on IFN-γ (a) and IL-10 expression (b) and IFN-γ to IL-10 expression ratio (c) in resident macrophage cells. Macrophage cells (1 × 10^6^/mL) isolated from peritoneal cavity were incubated with *Lactobacillus rhamnosus* NK210 (LR4, 1 × 10^4^ CFU/mL NK210; LR6, 1 × 10^5^ CFU/mL) or *Bifidobacterium longum* NK219 (BL4, 1 × 10^4^ CFU/mL NK210; BL6, 1 × 10^5^ CFU/mL) in the absence or presence of LPS. Normal control group (CON) was treated with saline instead of LPS. Data values were described as mean ± SD (n = 4). Data values indicate mean ± SD. ^#^*p* < 0.05 versus NC group. *p < 0.05 versus LP group.”

now reads:

“Effect of NK210 and NK219 on the expression of interferon (IFN)-γ and IL-10 in macrophages stimulated with or without LPS. (**A**) Effect on IFN-γ (a) and IL-10 expression (b) and IFN-γ to IL-10 expression ratio (c) in resident macrophage cells. (**B**) Effect on IFN-γ (a) and IL-10 expression (b) and IFN-γ to IL-10 expression ratio (c) in LPS-stimulated macrophage cells. Macrophage cells (1 × 10^6^/mL) isolated from peritoneal cavity were incubated with *Lactobacillus rhamnosus* NK210 (LR4, 1 × 10^4^ CFU/mL NK210; LR6, 1 × 10^5^ CFU/mL) or *Bifidobacterium longum* NK219 (BL4, 1 × 10^4^ CFU/mL NK210; BL6, 1 × 10^5^ CFU/mL) in the absence or presence of LPS. Normal control group (CON) was treated with saline instead of LPS. Data values were described as mean ± SD (n = 4). Data values indicate mean ± SD. ^#^*p* < 0.05 versus NC group. *p < 0.05 versus LP group.”

The original Article has been corrected.

