# Supplementary material for: Lactobacillus rhamnosus and Bifidobacterium longum alleviate colitis and cognitive impairment in mice by regulating IFN-γ to IL-10 and TNF-α to IL-10 expression ratios
Source: Sci Rep. 2021 Oct 19;11:20659. doi: 10.1038/s41598-021-00096-x (PMC8526673; doi:10.1038/s41598-021-00096-x)
Supplement: Supplementary file 1 — Supplementary Information. [file 41598_2021_96_MOESM1_ESM.docx]

**[Supplementary material]**

***Lactobacillus rhamnosus* and *Bifidobacterium longum* alleviate colitis and cognitive impairment in mice by regulating IFN-γ to IL-10 and TNF-α to IL-10 expression ratios**

Table S1. The gut microbiota composition of mice treated with NK210 (LR), NK219 (BL), and their combination (Mx) in the presence of ampicillin (AP) at the phylum level

| Taxon Name | Composition (%) | | | | |
| --- | --- | --- | --- | --- | --- |
|  | NC | AP | LR | BL | Mx |
| Bacteroidetes | 51.45±11.70 | 35.85±19.54 | 45.79±10.22 | 48.98±4.45 | 51.98±4.83 |
| Firmicutes | 41.84±12.15 | 24.87±15.47 | 22.82±3.52 | 23.76±6.75 | 29.16±13.90 |
| Cyanobacteria | 2.88±2.18 | 0.02±0.01**^#^** | 0.00±0.00 | 0.01±0.01 | 0.05±0.06 |
| Proteobacteria | 2.82±1.00 | 20.63±5.92**^#^** | 14.29±5.73 | 13.69±5.81 | 6.47±5.87***** |
| Verrucomicrobia | 0.72±0.70 | 18.58±5.38**^#^** | 16.69±5.20 | 13.36±6.63 | 11.62±6.88 |
| Tenericutes | 0.12±0.03 | 0.01±0.00**^#^** | 0.39±0.60 | 0.19±0.31 | 0.55±0.78 |
| Actinobacteria | 0.10±0.07 | 0.06±0.09 | 0.01±0.01 | 0.01±0.01 | 0.11±0.11 |
| Deferribacteres | 0.07±0.03 | 0.00±0.00**^#^** | 0.00±0.00 | 0.00±0.00 | 0.05±0.07 |
| Saccharibacteria_TM7 | 0.01±0.01 | 0.00±0.00 | 0.00±0.00 | 0.00±0.00 | 0.00±0.00 |

Values indicate mean ± SD. **^#^***p*<0.05 vs NC group and ******p*<0.05 vs  AP group.

Table S2. The gut microbiota composition of mice treated with NK210 (LR), NK219 (BL), and their combination (Mx) in the presence of ampicillin (AP) at the family level

| Taxon Name | Composition (%) | | | | |
| --- | --- | --- | --- | --- | --- |
|  | NC | AP | LR | BL | Mx |
| Lachnospiraceae | 28.02±9.83 | 15.38±9.59**^#^** | 19.30±4.21 | 19.77±5.43 | 21.67±11.73 |
| Muribaculaceae | 25.39±5.49 | 1.28±1.79**^#^** | 8.73±8.93 | 5.72±7.03 | 26.63±20.12***** |
| Prevotellaceae | 18.50±5.95 | 0.56±0.90**^#^** | 0.30±0.55 | 1.67±0.89 | 4.16±4.94 |
| Ruminococcaceae | 11.15±4.37 | 0.25±0.20**^#^** | 0.20±0.10 | 0.32±0.17 | 3.52±2.59***** |
| Bacteroidaceae | 3.95±2.33 | 30.50±16.14**^#^** | 10.80±9.64***** | 20.63±7.55 | 11.89±12.82 |
| FR888536_f | 2.88±2.18 | 0.02±0.01**^#^** | 0.00±0.00 | 0.01±0.01 | 0.05±0.06 |
| Rikenellaceae | 2.62±0.85 | 0.06±0.05**^#^** | 0.04±0.01 | 0.04±0.03 | 1.20±1.26 |
| Helicobacteraceae | 1.53±0.77 | 0.38±0.44**^#^** | 0.09±0.15 | 0.73±0.95 | 2.06±1.94 |
| Lactobacillaceae | 1.33±1.10 | 7.14±7.21 | 2.13±1.87 | 2.62±2.06 | 3.12±2.18 |
| Desulfovibrionaceae | 0.73±0.18 | 0.23±0.10**^#^** | 0.05±0.04***** | 0.19±0.23 | 0.24±0.16 |
| Akkermansiaceae | 0.72±0.70 | 18.58±5.38**^#^** | 16.69±5.20 | 13.36±6.63 | 11.62±6.88 |
| Porphyromonadaceae | 0.51±0.20 | 3.44±4.02 | 25.93±8.36***** | 20.92±3.67***** | 8.01±11.10 |
| Erysipelotrichaceae | 0.44±0.74 | 1.41±0.64**^#^** | 0.48±0.37***** | 0.54±0.39***** | 0.20±0.28***** |
| Christensenellaceae | 0.43±0.25 | 0.02±0.02**^#^** | 0.03±0.05 | 0.02±0.04 | 0.40±0.40***** |
| AC160630_f | 0.33±0.24 | 0.01±0.00**^#^** | 0.00±0.00 | 0.00±0.00 | 0.10±0.12 |
| Dehalobacterium_f | 0.31±0.12 | 0.01±0.00**^#^** | 0.00±0.00 | 0.00±0.00***** | 0.07±0.09 |
| Rhodospirillaceae | 0.24±0.20 | 0.00±0.00**^#^** | 0.00±0.00 | 0.00±0.00 | 0.28±0.39 |
| Sutterellaceae | 0.23±0.07 | 5.33±2.79**^#^** | 0.01±0.01***** | 0.01±0.01***** | 0.01±0.01***** |
| Odoribacteraceae | 0.10±0.06 | 0.00±0.00**^#^** | 0.00±0.00 | 0.00±0.00 | 0.00±0.00 |
| Coriobacteriaceae | 0.09±0.07 | 0.01±0.01**^#^** | 0.00±0.00 | 0.00±0.00 | 0.11±0.11***** |
| Enterobacterales_f | 0.07±0.01 | 13.29±5.65**^#^** | 12.06±5.79 | 11.84±4.80 | 3.33±4.95***** |
| Deferribacteraceae | 0.07±0.03 | 0.00±0.00**^#^** | 0.00±0.00 | 0.00±0.00 | 0.05±0.07 |
| Mogibacterium_f | 0.06±0.02 | 0.00±0.00**^#^** | 0.00±0.00 | 0.00±0.00 | 0.02±0.02 |
| Mycoplasmataceae | 0.06±0.014 | 0.01±0.00**^#^** | 0.39±0.00 | 0.19±0.31 | 0.52±0.78 |
| Clostridiaceae | 0.05±0.02 | 0.05±0.13 | 0.00±0.00 | 0.02±0.03 | 0.00±0.00 |

Values indicate mean ± SD. **^#^***p*<0.05 vs NC group and ******p*<0.05 vs  AP group.

Table S3. The gut microbiota composition of mice treated with NK210 (LR), NK219 (BL), and their combination (Mx) in the presence of ampicillin (AP) at the genus level

| Taxon Name | Composition (%) | | | | |
| --- | --- | --- | --- | --- | --- |
|  | NC | AP | LR | BL | Mx |
| Prevotellaceae_uc | 10.50±3.86 | 0.07±0.03**^#^** | 0.04±0.01 | 0.03±0.01***** | 0.46±0.68 |
| Prevotella | 7.56±2.96 | 0.04±0.03**^#^** | 0.01±0.00***** | 0.03±0.02 | 2.32±±3.32 |
| PAC001068_g | 6.99±1.33 | 0.11±0.02**^#^** | 5.77±7.44 | 3.68±4.97 | 7.36±5.26***** |
| PAC000186_g | 5.33±2.28 | 0.09±0.03**^#^** | 1.15±2.43 | 0.06±0.01 | 4.82±4.17***** |
| Bacteroides | 3.93±2.13 | 30.48±14.72**^#^** | 10.79±8.79***** | 20.61±6.88 | 11.89±11.70 |
| KE159538_g | 3.88±3.70 | 0.06±0.03**^#^** | 0.05±0.03 | 0.06±0.03 | 3.80±5.20 |
| Ruminococcus | 3.53±1.99 | 0.03±0.02**^#^** | 0.00±0.00***** | 0.01±0.00***** | 0.36±0.53 |
| PAC002367_g | 3.00±2.45 | 0.03±0.01**^#^** | 0.01±0.01 | 0.01±0.01***** | 0.01±0.01 |
| PAC000198_g | 2.92±1.04 | 0.04±0.01**^#^** | 0.04±0.03 | 0.19±0.38 | 2.61±1.94***** |
| FR888536_g | 2.88±1.99 | 0.02±0.01**^#^** | 0.00±0.00 | 0.01±0.00 | 0.05±0.06 |
| PAC001512_g | 2.46±0.79 | 0.02±0.01**^#^** | 0.01±0.00***** | 0.01±0.01 | 2.34±1.92***** |
| PAC000664_g | 2.35±1.84 | 0.04±0.01 | 0.03±0.02 | 0.08±0.07 | 2.58±2.31***** |
| Alistipes | 2.33±0.70 | 0.05±0.04**^#^** | 0.03±0.01 | 0.04±0.02 | 1.19±1.15 |
| PAC000661_g | 2.16±1.01 | 0.05±0.04**^#^** | 0.10±0.08 | 0.18±0.17 | 0.15±0.21 |
| PAC001472_g | 1.96±0.69 | 0.01±0.00**^#^** | 0.01±0.01 | 0.01±0.00 | 0.73±0.78 |
| PAC001228_g | 1.90±1.44 | 0.02±0.01**^#^** | 0.01±0.01 | 0.01±0.01 | 0.25±0.30 |
| LLKB_g | 1.83±2.22 | 0.02±0.01 | 0.02±0.01 | 0.02±0.01 | 0.47±0.40***** |
| Pseudoflavonifractor | 1.82±0.39 | 0.11±0.16**^#^** | 0.02±0.0.1 | 0.04±0.03 | 1.15±1.04 |
| Helicobacter | 1.53±0.70 | 0.38±0.40**^#^** | 0.09±0.14 | 0.73±0.87 | 2.06±1.77 |
| PAC001105_g | 1.52±2.07 | 0.01±0.01 | 0.01±0.01 | 0.01±0.01 | 0.01±0.01 |
| PAC001092_g | 1.37±1.21 | 0.01±0.01**^#^** | 0.01±0.01 | 0.01±0.01 | 1.05±0.88***** |
| Oscillibacter | 1.36±0.36 | 0.03±0.01**^#^** | 0.03±0.01 | 0.03±0.01 | 1.02±0.82***** |
| Lactobacillus | 1.32±1.00 | 7.14±6.58 | 2.13±1.70 | 2.61±1.88 | 3.12±1.99 |
| PAC000679_g | 0.90±0.28 | 0.00±0.00**^#^** | 0.00±0.00 | 0.00±0.00 | 0.04±0.04 |
| Eubacterium_g6 | 0.86±0.71 | 0.02±0.01**^#^** | 0.03±0.02 | 0.01±0.01 | 0.08±0.09 |

Values indicate mean ± SD. **^#^***p*<0.05 vs NC group and ******p*<0.05 vs  AP group.

Table S4. The gut microbiota composition of mice treated with NK210 (LR), NK219 (BL), and their combination (Mx) in the presence of ampicillin (AP) at the species level

| Taxon Name | Composition (%) | | | | |
| --- | --- | --- | --- | --- | --- |
|  | NC | AP | LR | BL | Mx |
| EU622763_s group | 4.07±2.49 | 4.07±0.02**^#^** | 4.07±0.00***** | 4.07±0.01 | 4.07±1.75 |
| PAC001065_s group | 3.56±1.07 | 3.56±0.02**^#^** | 3.56±0.01 | 3.56±0.01 | 3.56±2.40***** |
| PAC001696_s | 3.13±4.27 | 3.13±0.02 | 3.13±0.01***** | 3.13±0.00***** | 3.13±0.02 |
| DQ777952_s | 3.12±1.59 | 3.12±0.01**^#^** | 3.12±0.00 | 3.12±0.00 | 3.12±0.04 |
| PAC002367_s | 2.99±2.68 | 2.99±0.01**^#^** | 2.99±0.01 | 2.99±0.01***** | 2.99±0.01 |
| AB606236_s | 2.84±2.60 | 2.84±0.02**^#^** | 2.84±0.00***** | 2.84±0.01 | 2.84±0.01 |
| PAC001072_s | 2.47±0.76 | 2.47±0.01**^#^** | 2.47±0.01 | 2.47±0.01 | 2.47±0.94 |
| PAC001512_s | 2.26±0.86 | 2.26±0.01**^#^** | 2.26±0.00***** | 2.26±0.00***** | 2.26±0.00 |
| PAC000198_s | 2.07±1.10 | 2.07±0.01**^#^** | 2.07±0.01 | 2.07±0.01 | 2.07±1.66 |
| PAC001797_s | 2.04±1.97 | 2.04±0.01**^#^** | 2.04±0.00***** | 2.04±0.00 | 2.04±0.00***** |
| PAC001070_s group | 1.78±0.43 | 1.78±.0.01**^#^** | 1.78±0.01***** | 1.78±0.01 | 1.78±3.63 |
| EU622749_s | 1.73±0.71 | 1.73±0.01**^#^** | 1.73±0.00 | 1.73±0.00 | 1.73±0.16 |
| AB626939_s | 1.44±2.16 | 1.44±0.01 | 1.44±0.01 | 1.44±0.01 | 1.44±0.14***** |
| PAC001228_s | 1.35±1.70 | 1.35±0.00 | 1.35±0.01 | 1.35±0.00 | 1.35±0.00 |
| EF097112_s | 1.34±0.84 | 1.34±0.03**^#^** | 1.34±7.90 | 1.34±5.46 | 1.34±2.33 |
| AB599946_s | 1.28±1.02 | 1.28±0.56 | 1.28±8.54 | 1.28±4.16 | 1.28±7.01 |
| AB606242_s | 1.07±2.13 | 1.07±0.01 | 1.07±0.01 | 1.07±0.01 | 1.07±1.24 |
| Bacteroides acidifaciens group | 1.05±0.74 | 1.05±2.41 | 1.05±4.01 | 1.05±2.43 | 1.05±6.20 |
| Lactobacillus murinus group | 1.03±0.91 | 1.03±7.20 | 1.03±1.86 | 1.03±2.02 | 1.03±2.15 |
| PAC002445_s | 0.99±0.52 | 0.99±0.01**^#^** | 0.99±0.01 | 0.99±0.00 | 0.99±0.75***** |
| PAC001064_s | 0.98±0.87 | 0.98±0.01**^#^** | 0.98±2.64 | 0.98±0.01 | 0.98±2.33 |
| PAC001081_s group | 0.98±0.43 | 0.98±0.01**^#^** | 0.98±0.00 | 0.98±0.00 | 0.98±0.03 |
| PAC000679_s | 0.89±0.31 | 0.89±0.01**^#^** | 0.89±0.00 | 0.89±0.00 | 0.89±0.04 |
| Helicobacter japonicus | 0.89±0.44 | 0.89±0.01**^#^** | 0.89±0.01 | 0.89±0.03 | 0.89±1.00 |
| PAC001092_s | 0.85±1.43 | 0.85±0.00 | 0.85±0.00 | 0.85±0.00 | 0.85±0.36 |

Values indicate mean ± SD. **^#^***p*<0.05 vs NC group and ******p*<0.05 vs  AP group.

Table S5. The gut microbiota composition of mice treated with NK210 (LR), NK219 (BL), and their combination (Mx) in the presence of cyclophosphamide (CP) at the phylum level

| Taxon Name | Composition (%) | | | | |
| --- | --- | --- | --- | --- | --- |
|  | NC | CP | LR | BL | Mx |
| Bacteroidetes | 49.96±8.76 | 33.60±9.16**^#^** | 37.36±10.63 | 41.71±21.27 | 41.91±17.75 |
| Firmicutes | 44.87±9.82 | 61.33±9.61**^#^** | 58.73±10.35 | 47.71±2.39 | 52.86±18.29 |
| Tenericutes | 3.45±2.63 | 2.62±1.45 | 0.45±0.37***** | 0.90±0.58***** | 1.54±1.52 |
| Proteobacteria | 1.24±0.33 | 1.83±0.68 | 2.89±1.82 | 4.64±2.94***** | 2.74±1.30 |
| Actinobacteria | 0.21±0.16 | 0.19±0.10 | 0.15±0.06 | 0.16±0.07 | 0.24±0.14 |
| Cyanobacteria | 0.19±0.06 | 0.17±0.17 | 0.12±0.14 | 0.20±0.35 | 0.15±0.12 |
| Verrucomicrobia | 0.04±0.01 | 0.13±0.22 | 0.05±0.02 | 4.00±4.55 | 0.09±0.05 |
| Deferribacteres | 0.03±0.02 | 0.10±0.06 | 0.22±0.36 | 0.67±0.73 | 0.46±0.68 |
| Saccharibacteria_TM7 | 0.02±0.02 | 0.02±0.02 | 0.03±0.02 | 0.00±0.00 | 0.01±0.01 |

Values indicate mean ± SD. **^#^***p*<0.05 vs NC group and ******p*<0.05 vs  CP group.

Table S6. The gut microbiota composition of mice treated with NK210 (LR), NK219 (BL), and their combination (Mx) in the presence of cyclophosphamide (CP) at the family level

| Taxon Name | Composition (%) | | | | |
| --- | --- | --- | --- | --- | --- |
|  | NC | CP | LR | BL | Mx |
| Muribaculaceae | 30.91±7.24 | 19.07±6.55**^#^** | 20.89±8.65 | 29.33±15.27 | 25.11±12.38 |
| Lachnospiraceae | 19.93±6.59 | 32.77±9.91**^#^** | 41.65±15.92 | 34.78±18.48 | 37.22±15.12 |
| Lactobacillaceae | 18.07±13.05 | 18.35±9.58 | 7.20±6.92***** | 4.60±2.31***** | 3.14±2.51***** |
| Rikenellaceae | 6.86±4.73 | 3.60±0.70 | 3.60±1.69 | 3.66±1.28 | 3.03±1.22 |
| Prevotellaceae | 6.67±4.54 | 6.15±4.68 | 9.00±5.31 | 6.41±6.22 | 10.98±3.79 |
| Ruminococcaceae | 5.57±2.15 | 8.99±3.33 | 8.65±1.21 | 7.20±4.31 | 11.20±3.23 |
| Mycoplasmataceae | 3.33±2.67 | 2.54±1.44 | 0.33±0.39***** | 0.78±0.65 | 1.47±1.56 |
| Bacteroidaceae | 3.32±1.20 | 2.36±1.04 | 2.57±0.86 | 0.95±0.41***** | 1.83±1.16 |
| AC160630_f | 1.34±0.99 | 0.60±0.45 | 0.57±0.54 | 0.49±0.24 | 0.33±0.26 |
| Helicobacteraceae | 0.93±0.33 | 0.98±0.27 | 1.84±1.59 | 2.62±2.20 | 1.45±1.29 |
| Porphyromonadaceae | 0.55±0.13 | 0.32±0.14**^#^** | 0.29±0.09 | 0.47±0.24 | 0.55±0.30 |
| Clostridiaceae | 0.41±0.17 | 0.45±0.42 | 0.15±0.21 | 0.28±0.23 | 0.17±0.11 |
| Christensenellaceae | 0.36±0.15 | 0.35±0.18 | 0.45±0.31 | 0.45±0.23 | 0.47±0.24 |
| Odoribacteraceae | 0.29±0.50 | 1.47±0.59**^#^** | 0.40±0.40 | 0.39±0.40***** | 0.07±0.06***** |
| Coriobacteriaceae | 0.20±0.16 | 0.19±0.10 | 0.14±0.06 | 0.15±0.07 | 0.23±0.14 |
| Desulfovibrionaceae | 0.20±0.12 | 0.77±0.42**^#^** | 0.92±0.60 | 1.66±1.10 | 1.01±0.41 |
| FR888536_f | 0.19±0.06 | 0.17±0.17 | 0.12±0.14 | 0.20±0.35 | 0.15±0.12 |
| Enterococcaceae | 0.17±0.15 | 0.04±0.04 | 0.08±0.11 | 0.02±0.01 | 0.03±0.02 |
| Dehalobacterium_f | 0.17±0.15 | 0.22±0.11 | 0.30±0.16 | 0.23±0.14 | 0.40±0.11***** |
| Erysipelotrichaceae | 0.08±0.03 | 0.08±0.04 | 0.13±0.14 | 0.07±0.06 | 0.15±0.09 |
| Acholeplasmataceae | 0.06±0.06 | 0.00±0.01 | 0.00±0.00 | 0.04±0.06 | 0.03±0.05 |
| PAC000197_f | 0.05±0.05 | 0.04±0.03 | 0.10±0.06 | 0.03±0.01 | 0.03±0.02 |
| Sutterellaceae | 0.05±0.02 | 0.02±0.01**^#^** | 0.04±0.02***** | 0.07±0.06 | 0.05±0.04 |
| Mogibacterium_f | 0.05±0.02 | 0.04±0.02 | 0.06±0.01 | 0.04±0.01 | 0.07±0.03 |
| Akkermansiaceae | 0.04±0.01 | 0.13±0.22 | 0.05±0.02 | 4.00±4.55 | 0.09±0.05 |

Values indicate mean ± SD. **^#^***p*<0.05 vs NC group and ******p*<0.05 vs  CP group.

Table S7. The gut microbiota composition of mice treated with NK210 (LR), NK219 (BL), and their combination (Mx) in the presence of cyclophosphamide (CP) at the genus level

| Taxon Name | Composition (%) | | | | |
| --- | --- | --- | --- | --- | --- |
|  | NC | CP | LR | BL | Mx |
| Lactobacillus | 17.92±12.93 | 17.92±9.52 | 17.92±6.80***** | 17.92±2.30***** | 17.92±2.48***** |
| PAC001068_g | 10.28±4.40 | 10.28±1.38**^#^** | 10.28±1.52 | 10.28±3.53 | 10.28±4.23 |
| PAC000186_g | 6.64±1.45 | 6.64±2.50 | 6.64±3.26 | 6.64±4.48 | 6.64±4.36 |
| Alistipes | 5.66±4.50 | 5.66±0.94 | 5.66±1.60 | 5.66±1.53 | 5.66±1.41 |
| Prevotellaceae_uc | 4.14±3.06 | 4.14±1.50 | 4.14±4.96 | 4.14±4.29 | 4.14±3.51***** |
| Mycoplasma_g10 | 3.33±2.66 | 3.33±1.44 | 3.33±0.39***** | 3.33±0.65***** | 3.33±1.56 |
| Bacteroides | 3.32±1.20 | 3.32±1.04 | 3.32±0.85 | 3.32±0.41***** | 3.32±1.16 |
| KE159538_g | 3.15±1.88 | 3.15±2.58 | 3.15±9.85 | 3.15±3.31 | 3.15±3.58 |
| Muribaculum | 3.06±1.27 | 3.06±0.35**^#^** | 3.06±0.31 | 3.06±0.98 | 3.06±0.51 |
| PAC001127_g | 2.52±0.71 | 2.52±0.74**^#^** | 2.52±0.90 | 2.52±0.79 | 2.52±0.45 |
| PAC000198_g | 2.27±0.18 | 2.27±0.87 | 2.27±1.12 | 2.27±3.52 | 2.27±1.40 |
| PAC000664_g | 2.12±1.99 | 2.12±2.09 | 2.12±1.50 | 2.12±4.92 | 2.12±4.66 |
| Pseudoflavonifractor | 1.90±1.20 | 1.90±1.21 | 1.90±0.73 | 1.90±1.02 | 1.90±1.00 |
| Oscillibacter | 1.67±0.98 | 1.67±1.99 | 1.67±0.74 | 1.67±2.90 | 1.67±1.32 |
| PAC001765_g | 1.48±0.35 | 1.48±0.15**^#^** | 1.48±0.40 | 1.48±0.42 | 1.48±0.17 |
| Prevotella | 1.38±0.62 | 1.38±0.50 | 1.38±.32 | 1.38±1.10 | 1.38±1.09 |
| PAC001074_g | 1.35±0.72 | 1.35±0.57 | 1.35±0.42***** | 1.35±0.65 | 1.35±1.24 |
| PAC002482_g | 1.34±0.99 | 1.34±0.45 | 1.34±0.54 | 1.34±0.24 | 1.34±0.26 |
| KE159605_g | 1.16±1.02 | 1.16±0.17 | 1.16±0.26 | 1.16±0.32 | 1.16±.85 |
| PAC001112_g | 1.15±0.31 | 1.15±0.21**^#^** | 1.15±0.29 | 1.15±0.62 | 1.15±0.27 |
| Paraprevotella | 1.01±1.55 | 1.01±2.16 | 1.01±0.40 | 1.01±2.02 | 1.01±0.01 |
| PAC001228_g | 0.98±0.65 | 0.98±1.14 | 0.98±2.28 | 0.98±1.37 | 0.98±0.54 |
| Helicobacter | 0.93±0.33 | 0.93±0.27 | 0.93±1.59 | 0.93±2.20 | 0.93±1.28 |
| PAC002367_g | 0.93±1.17 | 0.93±0.67 | 0.93±1.13 | 0.93±0.03 | 0.93±0.14 |
| LLKB_g | 0.84±0.37 | 0.84±1.35 | 0.84±3.95 | 0.84±3.53 | 0.84±1.19 |

Values indicate mean ± SD. **^#^***p*<0.05 vs NC group and ******p*<0.05 vs  CP group.

Table S8. The gut microbiota composition of mice treated with NK210 (LR), NK219 (BL), and their combination (Mx) in the presence of cyclophosphamide (CP) at the species level

| Taxon Name | Composition (%) | | | | |
| --- | --- | --- | --- | --- | --- |
|  | NC | CP | LR | BL | Mx |
| PAC001065_s group | 4.42±1.33 | 3.66±1.40 | 6.18±1.90 | 4.30±2.75 | 5.18±3.38 |
| PAC002444_s | 4.33±4.07 | 0.24±0.23 | 0.26±0.39 | 0.45±0.44 | 0.97±0.23 |
| PAC001072_s | 3.34±1.53 | 0.43±0.23 | 0.30±0.26 | 1.18±0.97 | 1.25±0.80 |
| Mycoplasma sualvi | 3.25±2.57 | 2.50±1.42 | 0.33±0.39 | 0.78±0.65 | 1.46±1.54 |
| PAC002399_s | 2.51±0.71 | 1.46±0.74**^#^** | 1.36±0.90 | 2.07±0.78 | 0.85±0.45 |
| PAC001070_s group | 2.29±1.43 | 0.33±0.19**^#^** | 0.01±0.01***** | 0.09±0.07***** | 0.12±0.11***** |
| PAC001077_s | 2.03±1.12 | 0.25±0.15**^#^** | 0.13±0.08 | 0.48±0.25 | 0.20±0.15 |
| AB599946_s | 1.85±0.92 | 0.56±0.79**^#^** | 0.78±0.70 | 0.36±0.28 | 0.90±0.65 |
| PAC001696_s | 1.77±2.23 | 1.50±2.34 | 3.53±4.21 | 0.03±0.03 | 1.35±3.22 |
| PAC001064_s | 1.67±0.32 | 1.70±1.05 | 1.58±0.89 | 2.01±1.37 | 0.68±±0.52 |
| PAC001139_s | 1.59±0.27 | 1.41±0.53 | 0.65±0.32***** | 0.71±0.22***** | 0.71±0.37***** |
| EF097112_s | 1.52±0.36 | 0.75±0.36**^#^** | 1.04±0.32 | 1.37±0.96 | 2.02±1.06 |
| Lactobacillus reuteri group | 1.50±0.83 | 1.19±0.90 | 2.64±2.87 | 1.22±0.99 | 0.83±0.73***** |
| PAC001071_s | 1.39±0.73 | 1.18±0.72 | 1.07±0.83 | 1.25±0.48 | 0.75±0.36 |
| EF603735_s | 1.16±0.99 | 0.44±0.42 | 0.47±0.48 | 0.36±0.23 | 0.20±0.23 |
| PAC001074_s | 1.16±0.54 | 1.58±0.54 | 0.65±0.38***** | 1.13±0.62 | 2.66±1.21 |
| PAC002401_s group | 1.10±1.07 | 0.42±0.25 | 0.70±0.26 | 0.02±0.01***** | 0.04±0.03***** |
| FJ880724_s | 1.01±1.54 | 1.16±2.15 | 0.22±0.41 | 1.35±2.02 | 0.01±0.01 |
| PAC002451_s | 1.00±0.25 | 0.31±0.15**^#^** | 0.22±0.16 | 0.22±0.23 | 0.13±0.09***** |
| PAC002367_s | 0.93±1.17 | 0.51±0.67 | 1.18±1.12 | 0.02±0.03 | 0.13±0.14 |
| Muribaculum intestinale | 0.90±0.28 | 0.35±0.13**^#^** | 0.28±0.09 | 0.91±0.54***** | 0.48±0.29 |
| PAC002450_s | 0.88±0.57 | 0.23±0.13**^#^** | 0.12±0.07 | 0.98±0.84 | 1.33±1.02***** |
| PAC002481_s | 0.80±0.24 | 0.32±0.24**^#^** | 0.06±0.05***** | 0.09±0.07***** | 0.17±0.20 |
| PAC001066_s | 0.76±0.24 | 1.00±0.61 | 0.55±0.48 | 0.96±0.61 | 0.20±0.14***** |

Values indicate mean ± SD. **^#^***p*<0.05 vs NC group and ******p*<0.05 vs  CP group.

Table S9. The gut microbiota composition of mice treated with NK210 (LR), NK219 (BL), and their combination (Mx) at the phylum level

| Taxon Name | Composition (%) | | | |
| --- | --- | --- | --- | --- |
|  | NC | LR | BL | Mx |
| Firmicutes | 58.26±15.22 | 57.02±9.68 | 51.74±19.20 | 60.27±16.71 |
| Bacteroidetes | 35.00±15.05 | 38.75±10.23 | 42.15±21.80 | 34.59±18.02 |
| Proteobacteria | 3.87±1.10 | 2.30±1.16**^#^** | 3.85±4.44 | 3.44±1.99 |
| Tenericutes | 2.25±2.46 | 1.17±0.98 | 1.48±1.65 | 0.87±0.38 |
| Actinobacteria | 0.36±0.28 | 0.42±0.18 | 0.32±0.21 | 0.55±0.46 |
| Cyanobacteria | 0.11±0.08 | 0.10±0.06 | 0.32±0.55 | 0.11±0.10 |
| Verrucomicrobia | 0.07±0.04 | 0.04±0.01 | 0.05±0.02 | 0.04±0.02 |
| Deferribacteres | 0.05±0.06 | 0.18±0.31 | 0.08±0.07 | 0.12±0.11 |
| Saccharibacteria_TM7 | 0.02±0.02 | 0.02±0.01 | 0.01±0.01 | 0.02±0.01 |

Values indicate mean ± SD. **^#^***p*<0.05 vs NC group

Table S10. The gut microbiota composition of mice treated with NK210 (LR), NK219 (BL), and their combination (Mx) at the family level

| Taxon Name | Composition (%) | | | |
| --- | --- | --- | --- | --- |
|  | NC | LR | BL | Mx |
| Lachnospiraceae | 42.36±13.54 | 40.35±12.17 | 36.01±15.94 | 43.94±14.84 |
| Muribaculaceae | 14.83±4.62 | 20.48±7.75 | 24.52±11.76 | 21.84±11.96 |
| Rikenellaceae | 9.10±4.25 | 4.55±2.07**^#^** | 2.50±1.94**^#^** | 2.82±2.17**^#^** |
| Ruminococcaceae | 8.92±3.74 | 9.65±2.68 | 9.37±4.17 | 10.34±3.85 |
| Prevotellaceae | 7.72±8.16 | 8.94±6.54 | 10.17±12.56 | 4.93±3.31 |
| Lactobacillaceae | 5.55±2.90 | 6.11±5.13 | 5.47±4.02 | 5.09±2.45 |
| Helicobacteraceae | 2.83±1.23 | 1.35±0.94**^#^** | 2.92±4.27 | 2.41±1.99 |
| Mycoplasmataceae | 2.18±2.45 | 1.07±1.01 | 1.40±1.63 | 0.82±0.40 |
| Bacteroidaceae | 1.62±1.23 | 2.97±2.09 | 2.82±1.10 | 3.69±3.32 |
| Desulfovibrionaceae | 0.96±0.44 | 0.85±0.47 | 0.79±0.38 | 0.94±0.25 |
| Odoribacteraceae | 0.84±0.85 | 0.50±0.30 | 0.57±0.76 | 0.19±0.07 |
| Christensenellaceae | 0.51±0.42 | 0.21±0.06 | 0.37±0.25 | 0.30±0.11 |
| Porphyromonadaceae | 0.50±0.31 | 0.61±0.19 | 0.79±0.30 | 0.48±0.33 |
| AC160630_f | 0.38±0.45 | 0.68±0.56 | 0.76±0.66 | 0.64±0.63 |
| Coriobacteriaceae | 0.36±0.28 | 0.42±0.18 | 0.32±0.21 | 0.54±0.46 |
| Dehalobacterium_f | 0.34±0.12 | 0.28±0.20 | 0.22±0.13 | 0.21±0.06 |
| Erysipelotrichaceae | 0.26±0.29 | 0.09±0.05 | 0.06±0.02 | 0.09±0.09 |
| Clostridiaceae | 0.20±0.15 | 0.10±0.07 | 0.13±0.07 | 0.13±0.08 |
| FR888536_f | 0.11±0.08 | 0.10±0.06 | 0.32±0.55 | 0.11±0.10 |
| Akkermansiaceae | 0.07±0.04 | 0.04±0.01 | 0.05±0.02 | 0.04±0.02 |
| Enterococcaceae | 0.05±0.02 | 0.11±0.11 | 0.05±0.03 | 0.04±0.03 |
| Deferribacteraceae | 0.05±0.06 | 0.18±0.31 | 0.08±0.07 | 0.12±0.11 |
| Mogibacterium_f | 0.05±0.03 | 0.07±0.04 | 0.03±0.02 | 0.08±0.03 |
| Sutterellaceae | 0.04±0.04 | 0.04±0.01 | 0.04±0.04 | 0.04±0.03 |
| PAC000197_f | 0.03±0.02 | 0.05±0.05 | 0.03±0.03 | 0.03±0.02 |

Values indicate mean ± SD. **^#^***p*<0.05 vs NC group

Table S11. The gut microbiota composition of mice treated with NK210 (LR), NK219 (BL), and their combination (Mx) at the genus level

| Taxon Name | Composition (%) | | | |
| --- | --- | --- | --- | --- |
|  | NC | LR | BL | Mx |
| KE159538_g | 12.87±15.20 | 3.33±1.62 | 3.87±3.53 | 3.99±1.85 |
| Alistipes | 7.79±3.86 | 3.61±2.00**^#^** | 1.78±1.95**^#^** | 1.69±2.03**^#^** |
| Prevotellaceae_uc | 5.75±6.41 | 6.29±3.95 | 8.52±10.73 | 4.54±2.90 |
| Lactobacillus | 5.53±2.89 | 6.10±5.12 | 5.43±3.99 | 5.06±2.44 |
| PAC001068_g | 5.19±1.78 | 5.65±2.47 | 7.71±3.78 | 4.62±2.75 |
| PAC000664_g | 3.43±0.90 | 6.14±3.84 | 4.48±1.24 | 2.85±1.30 |
| Oscillibacter | 3.17±1.96 | 4.05±1.99 | 3.29±1.74 | 4.39±2.25 |
| Pseudoflavonifractor | 2.93±1.25 | 2.59±0.90 | 3.33±2.04 | 3.18±1.15 |
| Helicobacter | 2.83±1.23 | 1.35±0.94 | 2.92±4.27 | 2.41±1.99 |
| PAC000186_g | 2.42±1.01 | 5.05±2.57**^#^** | 7.53±3.90**^#^** | 8.86±4.88**^#^** |
| PAC000198_g | 2.20±1.14 | 1.73±0.77 | 1.50±0.89 | 1.76±1.28 |
| Mycoplasma_g10 | 2.18±2.44 | 1.07±1.01 | 1.40±1.63 | 0.82±0.40 |
| PAC000692_g | 1.83±1.21 | 0.72±0.79 | 0.73±0.82 | 0.60±0.26**^#^** |
| KE159605_g | 1.73±1.88 | 2.80±3.25 | 0.47±0.33 | 0.99±1.03 |
| PAC001228_g | 1.73±0.94 | 0.78±0.30**^#^** | 1.35±0.89 | 1.26±0.76 |
| PAC002367_g | 1.68±3.04 | 2.30±1.32 | 2.29±3.29 | 2.97±1.62 |
| LLKB_g | 1.65±0.81 | 1.15±1.00 | 1.84±1.36 | 3.86±3.63 |
| PAC001127_g | 1.62±0.77 | 0.94±0.55 | 1.30±0.84 | 1.15±0.48 |
| Bacteroides | 1.62±1.23 | 2.96±2.09 | 2.82±1.10 | 3.69±3.32 |
| Paraprevotella | 1.15±1.65 | 0.36±0.56 | 0.03±0.02 | 0.13±0.12 |
| PAC001105_g | 1.09±1.77 | 0.11±0.05 | 1.48±2.12 | 1.55±1.76 |
| PAC001043_g | 1.08±0.51 | 0.79±0.29 | 0.66±0.48 | 0.77±0.32 |
| PAC001092_g | 0.99±0.44 | 1.13±0.47 | 1.12±0.74 | 0.95±0.53 |
| HM630235_g | 0.99±0.52 | 0.53±0.24 | 0.34±0.14**^#^** | 0.60±0.25 |
| PAC001097_g | 0.97±1.42 | 0.09±0.10 | 0.04±0.09 | 0.34±0.58 |

Values indicate mean ± SD. **^#^***p*<0.05 vs NC group

Table S12. The gut microbiota composition of mice treated with NK210 (LR), NK219 (BL), and their combination (Mx) at the species level

| Taxon Name | Composition (%) | | | |
| --- | --- | --- | --- | --- |
|  | NC | LR | BL | Mx |
| KE159538_g_uc | 9.44±16.21 | 0.13±0.08 | 1.69±4.01 | 0.04±0.02 |
| PAC002444_s | 5.61±3.00 | 2.18±1.56**^#^** | 0.97±1.56**^#^** | 0.62±0.095**^#^** |
| Lactobacillus murinus group | 4.56±3.08 | 5.71±4.92 | 4.34±2.88 | 4.32±2.22 |
| Mycoplasma sualvi | 2.16±2.42 | 1.06±0.99 | 1.39±1.62 | 0.81±0.40 |
| PAC001070_s group | 2.03±1.09 | 0.54±0.29**^#^** | 1.48±1.14 | 0.32±0.32**^#^** |
| PAC002367_s | 1.68±3.03 | 2.30±1.32 | 2.29±3.28 | 2.96±1.61 |
| Helicobacter rodentium group | 1.65±1.36 | 0.86±0.62 | 1.82±2.70 | 1.86±2.06 |
| PAC002399_s | 1.62±0.77 | 0.94±0.55 | 1.29±0.84 | 1.14±0.48 |
| PAC001072_s | 1.54±1.04 | 1.98±1.12 | 3.34±1.98 | 1.90±1.34 |
| PAC001065_s group | 1.48±0.99 | 3.23±1.85 | 2.01±1.13 | 2.60±1.24 |
| PAC001770_s | 1.44±1.96 | 0.02±0.01 | 0.02±0.04 | 0.36±00.83 |
| PAC000198_s | 1.42±0.85 | 0.06±0.01**^#^** | 0.39±0.40 | 0.67±0.54**^#^** |
| PAC002401_s group | 1.28±2.07 | 2.61±3.26 | 0.02±0.01 | 0.44±00.74 |
| PAC001094_s | 1.24±2.06 | 0.09±0.07 | 0.05±0.10 | 0.08±0.14 |
| FJ880724_s | 1.15±1.65 | 0.36±0.56 | 0.02±0.02 | 0.13±0.12 |
| Helicobacter japonicus | 1.14±0.85 | 0.47±0.37 | 1.02±1.43 | 0.52±0.35 |
| PAC001060_s | 1.11±0.83 | 0.45±0.28 | 0.08±0.08**^#^** | 0.46±0.87 |
| EU791148_s | 0.99±0.52 | 0.53±0.24 | 0.34±0.14**^#^** | 0.60±0.25 |
| AB626939_s | 0.95±0.61 | 0.25±0.29**^#^** | 1.13±0.98 | 2.56±3.05 |
| PAC001097_s | 0.95±1.40 | 0.08±0.10 | 0.04±0.09 | 0.34±0.58 |
| AB606242_s | 0.84±0.97 | 1.43±1.09 | 0.58±0.83 | 0.63±0.71 |
| AB599946_s | 0.81±0.66 | 2.25±1.99 | 1.85±0.93 | 2.64±2.54 |
| AB606328_s | 0.81±0.84 | 0.46±0.30 | 0.54±0.74 | 0.16±0.06 |
| PAC000692_g_uc | 0.79±0.64 | 0.46±0.72 | 0.30±0.52 | 0.27±0.17 |
| AB606285_s | 0.78±1.69 | 0.04±0.04 | 0.54±0.83 | 0.75±1.47 |

Values indicate mean ± SD. **^#^***p*<0.05 vs NC group

Table S13. Primer sequences used in the present study

| Gene | Primer | Sequence |
| --- | --- | --- |
| Foxp3 | Forward | 5’-AGAAGCTGGGAGCTATGCAG-3’ |
|  | Reverse | 5’-GCTACGATGCAGCAAGCGC-3’ |
| Tbet | Forward | 5’-TGCCCGAACTACAGTCACGAAC-3’ |
|  | Reverse | 5’-AGTGACCTCGCCTGGTGAAATG-3’ |
| GAPDH | Forward | 5’-TGCAGTGGCAAAGTGGAGAT-3’ |
|  | Reverse | 5’-TTTGCCGTGAGTGGAGTCATA-3’ |

(a)


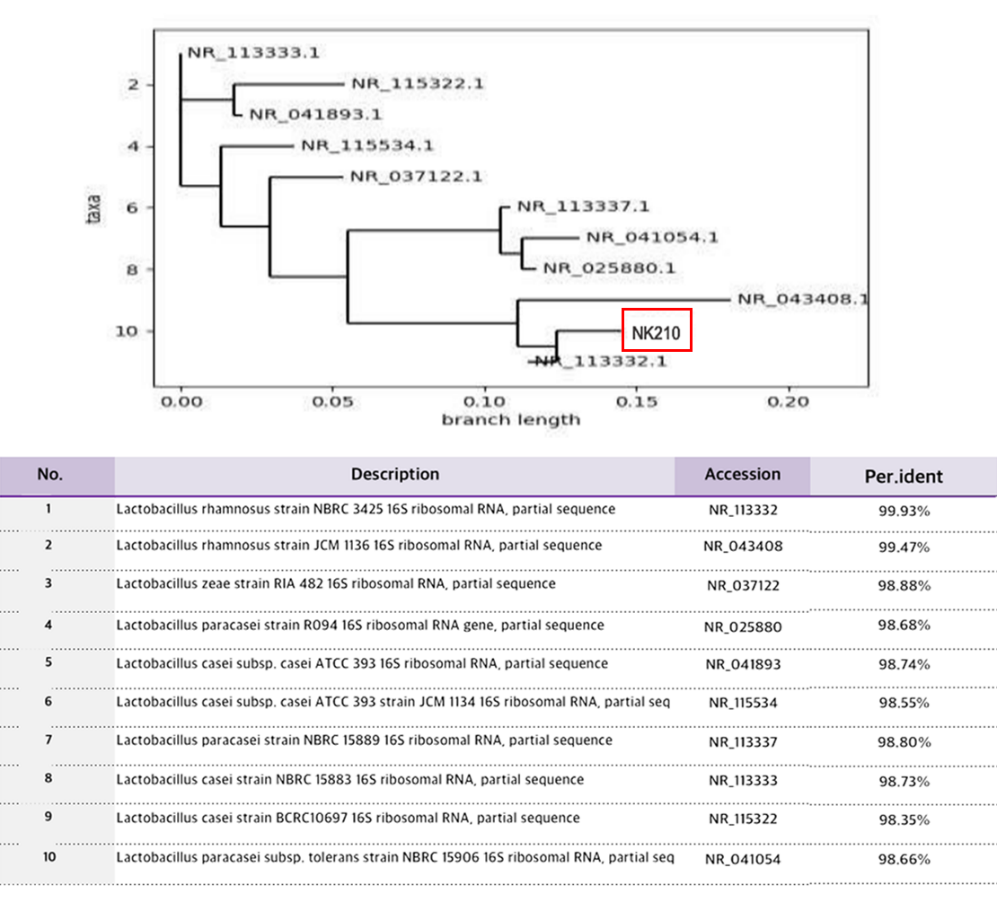


(b)


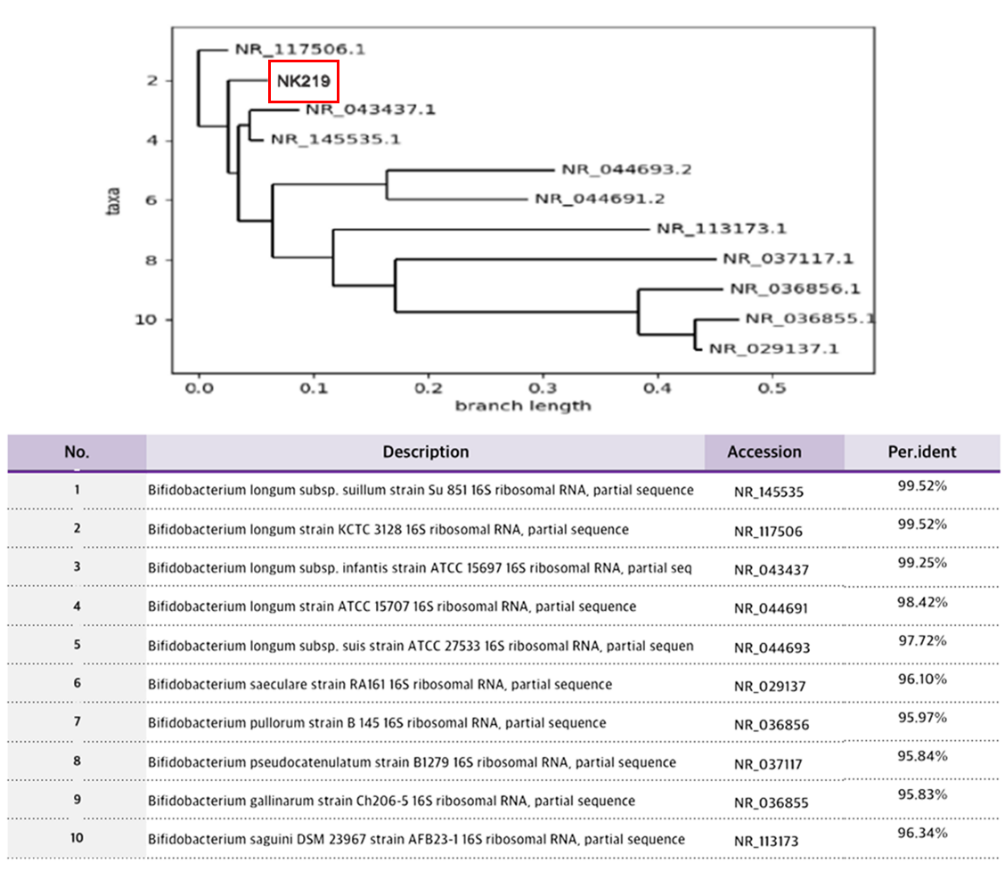


Figure S1. Characteristics of NK210 and NK219. The phylogenetic trees of *Lactobacillus rhamnosus* (a) and *Bifidobacterium longum* (b) based on the 16S rRNA.


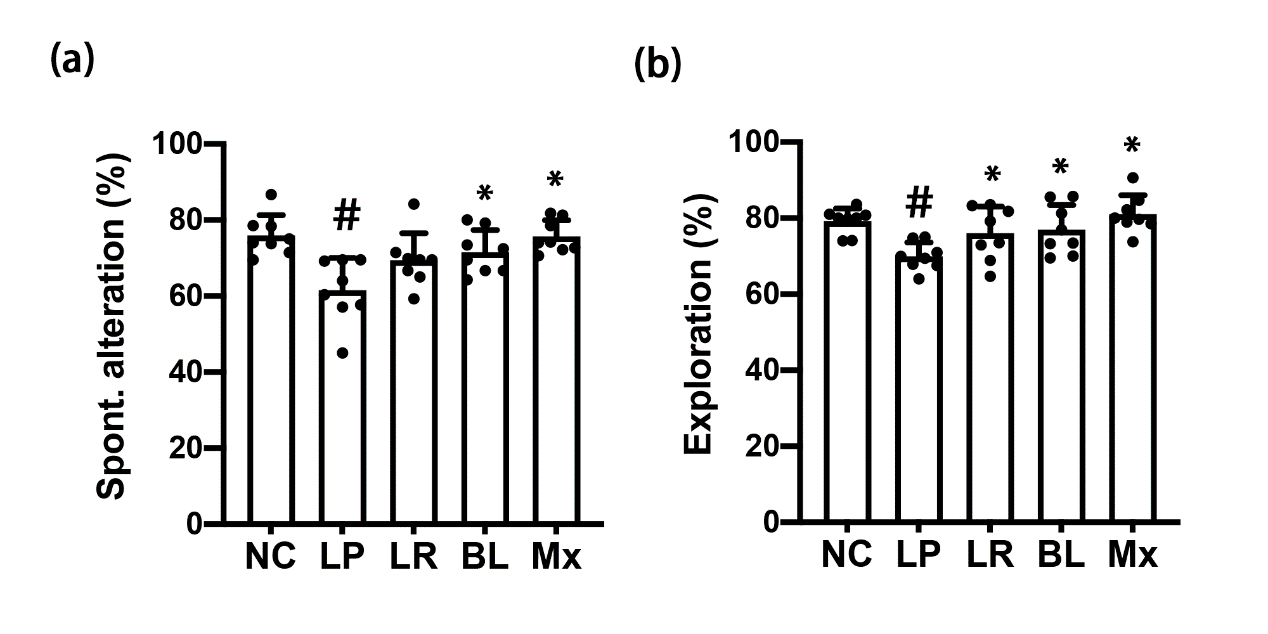


Figure S2. Effects of NK210 and NK219 on LPS-induced immune imbalance in mice. Effects on cognitive impairment in the Y-maze (a) and NOR task (b). Test agents (LP, LPS alone; LR, 1×10^9^ CFU/mouse/day of NK210; BL, 1×10^9^ CFU/mouse/day of NK219; Mx, 1×10^9^ CFU/mouse/day of LR and BL [4:1] mix) were orally gavaged daily for 5 days after intraperitoneal injection of LPS. Normal control mice (NC) were treated with vehicle (saline) instead of test agents. Cytokine levels were assayed by using ELISA kits. Data values indicate mean ± SD (n=8). ^#^p<0.05 vs NC group. *p<0.05 vs LP group treated with LPS alone.


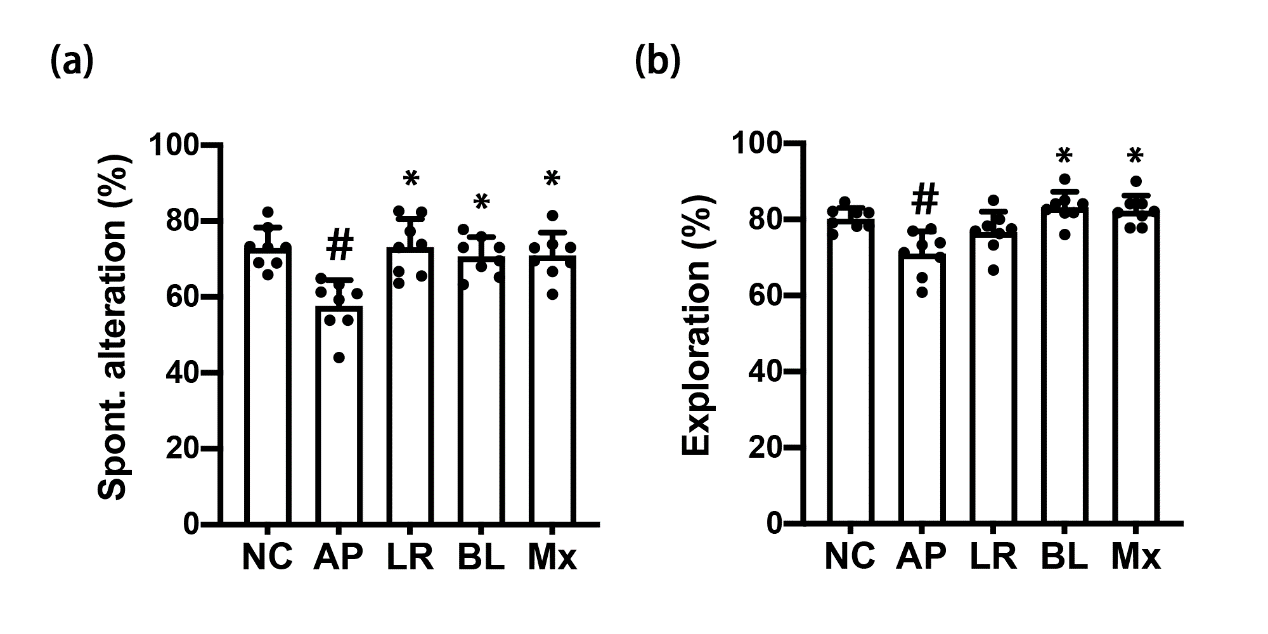


Figure S3. Effects of NK210 and NK219 on ampicillin-induced immune imbalance in mice. Effects on cognitive impairment in the Y-maze (a) and NOR task (b). Test agents (AP, ampicillin alone; LR, 1×10^9^ CFU/mouse/day of NK210; BL, 1×10^9^ CFU/mouse/day of NK219; Mx, 1×10^9^ CFU/mouse/day of LR and BL [4:1] mix) were orally gavaged daily for 5 days after oral gavage of ampicillin. Normal control mice (NC) were treated with vehicle (saline) instead of test agents. Data values indicate mean ± SD (n=8). ^#^p<0.05 vs NC group. *p<0.05 vs AP group treated with ampicillin alone.


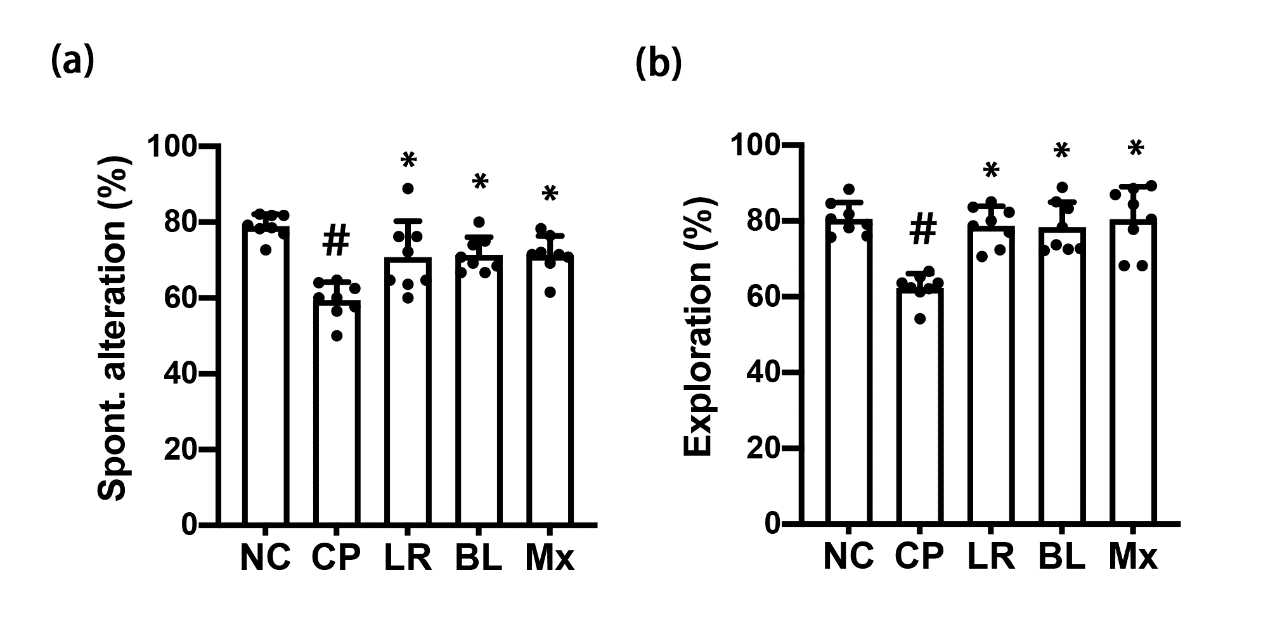


Figure S4. Effects of NK210 and NK219 on cyclophosphamide-induced immune imbalance in mice. Effects on cognitive impairment in the Y-maze (a) and NOR task (b). Test agents (CP, cyclophosphamide alone; LR, 1×10^9^ CFU/mouse/day of NK210; BL, 1×10^9^ CFU/mouse/day of NK219; Mx, 1×10^9^ CFU/mouse/day of LR and BL [4:1] mix) were orally gavaged daily for 5 days after intraperitoneal injection of cyclophosphamide. Normal control mice (NC) were treated with vehicle (saline) instead of test agents. Data values indicate mean ± SD (n=8). ^#^p<0.05 vs NC group. *p<0.05 vs CP group treated with cyclophosphamide alone.


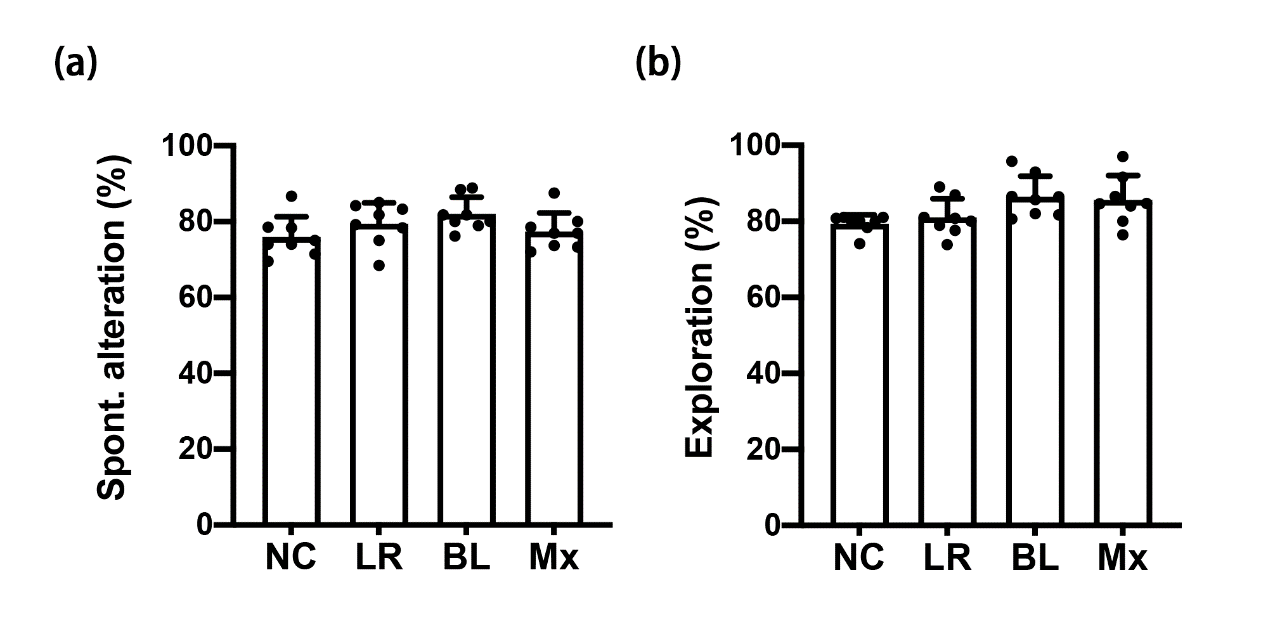


Figure S5. Effects of NK210 and NK219 on the immune balance in mice. Effects on cognitive impairment in the Y-maze (a) and NOR task (b). Test agents (LR, 1×10^9^ CFU/mouse/day of NK210; BL, 1×10^9^ CFU/mouse/day of NK219; Mx, 1×10^9^ CFU/mouse/day of LR and BL [4:1] mix) were orally gavaged daily for 5 days. Normal control mice (NC) were treated with vehicle (saline) instead of test agents. Cytokine levels were assayed by using ELISA kits. Data values indicate mean ± SD (n=8). ^#^p<0.05 vs NC group.


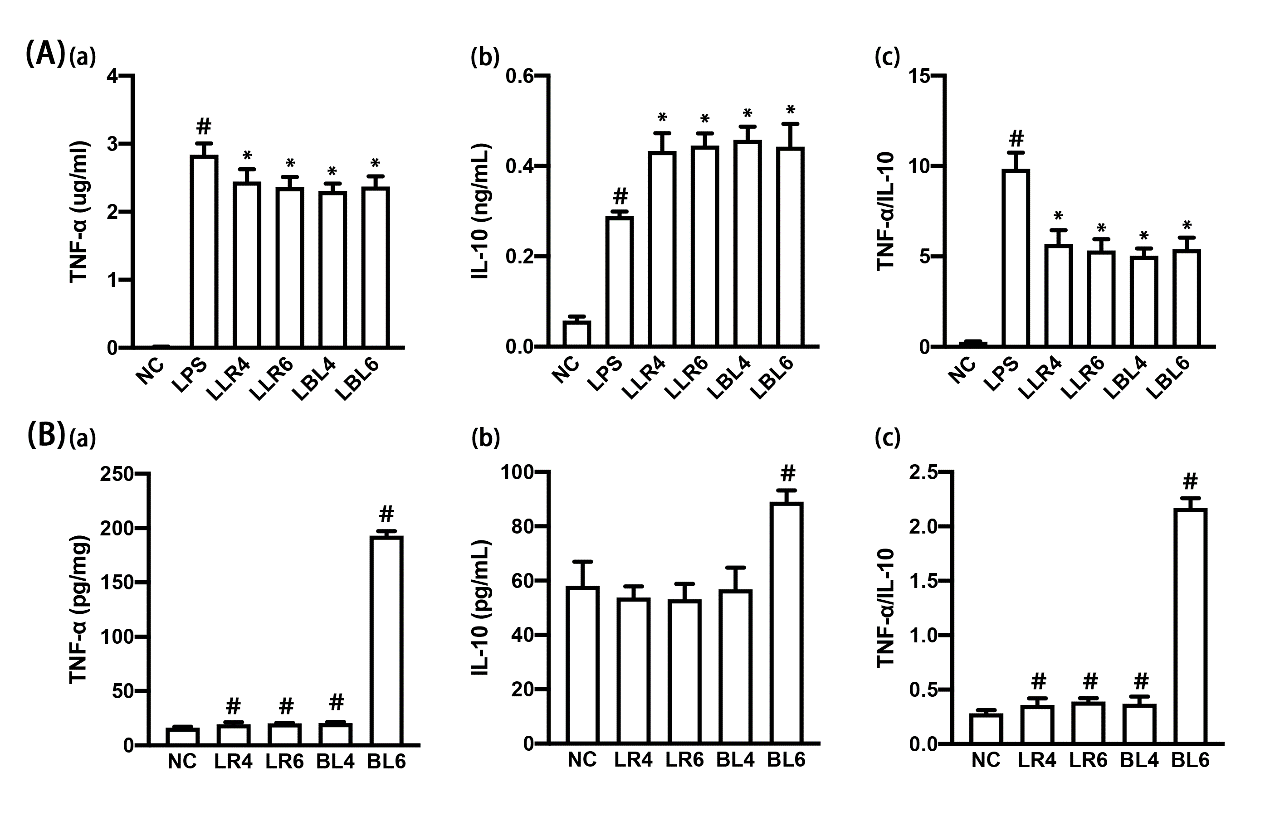


Figure S6. Effect of NK210 and NK219 on the expression of TNF-α and IL-10 in macrophages stimulated with or without LPS. (A) Effect on TNF-α (a) and IL-10 expression (b) and TNF-α to IL-10 expression ratio (c) in LPS-stimulated macrophage cells. (B) Effect on TNF-α (a) and IL-10 expression (b) and TNF-α to IL-10 expression ratio (c) in resident macrophage cells. Macrophage cells (1 × 10^6^/mL) isolated from peritoneal cavity were incubated with *Lactobacillus rhamnosus* NK210 (LR4, 1 × 10^4^ CFU/mL NK210; LR6, 1 × 10^5^ CFU/mL) or *Bifidobacterium longum* NK219 (BL4, 1 × 10^4^ CFU/mL NK210; BL6, 1 × 10^5^ CFU/mL) in the absence or presence of LPS. Normal control group (CON) was treated with saline instead of LPS. Data values were described as mean ± SD (n = 4). Data values indicate mean ± SD. ^#^p<0.05 vs NC group. *p<0.05 vs LP group.


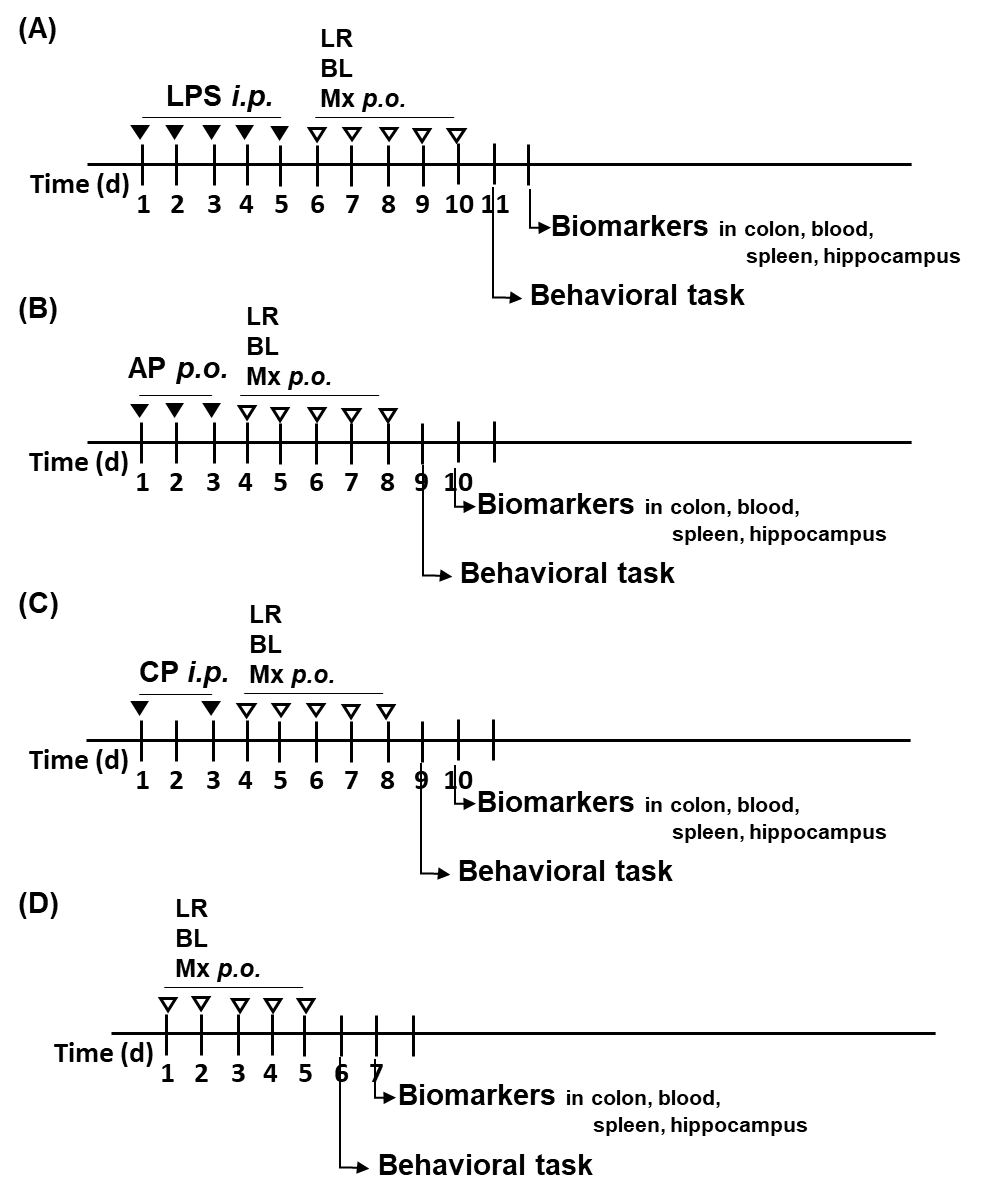


Figure S7. Experimental schedules. (a) A study on the effects of NK210 (LR), NK219 (BL), and their mixture (Mx) in mice with LPS-induced systemic inflammation. (b) A study on the effects of NK210 (LR), NK219 (BL), and their mixture (Mx) in mice with ampicillin-induced gut dysbiosis. (c) A study on the effects of NK210 (LR), NK219 (BL), and their mixture (Mx) in normal control mice.
